# Assessing the quality of evidence in studies estimating prevalence of exposure to occupational risk factors: The QoE-SPEO approach applied in the systematic reviews from the WHO/ILO Joint Estimates of the Work-related burden of disease and Injury

**DOI:** 10.1016/j.envint.2022.107136

**Published:** 2022-03

**Authors:** Frank Pega, Natalie C. Momen, Diana Gagliardi, Lisa A. Bero, Fabio Boccuni, Nicholas Chartres, Alexis Descatha, Angel M. Dzhambov, Lode Godderis, Tom Loney, Daniele Mandrioli, Alberto Modenese, Henk F. van der Molen, Rebecca L. Morgan, Subas Neupane, Daniela Pachito, Marilia S. Paulo, K.C. Prakash, Paul T.J. Scheepers, Liliane Teixeira, Thomas Tenkate, Tracey J. Woodruff, Susan L. Norris

**Affiliations:** a Department of Environment, Climate Change and Health, World Health Organization, Geneva, Switzerland; b Inail, Department of Occupational and Environmental Medicine, Epidemiology and Hygiene, Rome, Italy; c Charles Perkins Centre, The University of Sydney, Sydney, Australia; d General Internal Medicine/Public Health/Center for Bioethics and Humanities, University of Colorado—Anschutz Medical Campus, Denver, CO, United States; e Program on Reproductive Health and the Environment, Department of Obstetrics, Gynecology and Reproductive Sciences, University of California San Francisco, San Francisco, United States; f AP-HP (Paris Hospital “Assistance Publique Hôpitaux de Paris”), Occupational Health Unit, University Hospital of West Suburb of Paris, Poincaré Site, Garches, France /Versailles St-Quentin Univ - Paris Saclay Univ (UVSQ), UMS 011, UMR-S 1168, France; g Univ Angers, CHU Angers, Univ Rennes, Inserm, EHESP, Irset (Institut de recherche en santé, environnement et travail) - UMR_S1085, CAPTV CDC, Angers, France; h Department of Hygiene, Faculty of Public Health, Medical University of Plovdiv, Plovdiv, Bulgaria; i Institute for Highway Engineering and Transport Planning, Graz University of Technology, Graz, Austria; j Centre for Environment and Health, KU Leuven, Leuven, Belgium; k KIR Department (Knowledge, Information & Research), IDEWE, External Service for Prevention and Protection at Work, Leuven, Belgium; l College of Medicine, Mohammed Bin Rashid University of Medicine and Health Sciences, Dubai, United Arab Emirates; m Cesare Maltoni Cancer Research Center, Ramazzini Institute, Bologna, Italy; n Department of Biomedical, Metabolic and Neural Sciences, University of Modena and Reggio Emilia, Modena, Italy; o Coronel Institute of Occupational Health, Amsterdam UMC, location AMC, Amsterdam Public Health Research Institute, Amsterdam, Netherlands; p Department of Health Research Methods, Evidence and Impact, McMaster University, Ontario, Canada; q Faculty of Social Science (Health Sciences), University of Tampere, Tampere, Finland; r Evidence-based Health, Universidade Federal de São Paulo, Sao Paulo, Brazil; s Cochrane Brazil, Sao Paulo, Brazil; t Institute of Public Health, College of Medicine & Health Sciences, United Arab Emirates University, Al Ain, United Arab Emirates; u Global Health and Tropical Medicine, Instituto de Higiene e Medicina Tropical, Universidade Nova de Lisboa, Lisbon, Portugal; v Radboud Institute for Health Sciences, Radboudumc, Nijmegen, the Netherlands; w Workers' Health and Human Ecology Research Center, National School of Public Health Sergio Arouca, Oswaldo Cruz Foundation, Rio de Janeiro, RJ, Brazil; x School of Occupational and Public Health, Ryerson University, Toronto, Ontario, Canada; y Oregon Health & Science University, Portland, OR, United States; z Department of Quality Assurance, Norms and Standards, World Health Organization, Geneva, Switzerland

**Keywords:** Quality of evidence, Systematic review, Prevalence studies, Exposure science, Occupational health, Body of evidence

## Abstract

**Background:**

The World Health Organization (WHO) and the International Labour Organization (ILO) have produced the WHO/ILO Joint Estimates of the Work-related Burden of Disease and Injury (WHO/ILO Joint Estimates). For these, systematic reviews of studies estimating the prevalence of exposure to selected occupational risk factors have been conducted to provide input data for estimations of the number of exposed workers. A critical part of systematic review methodology is to assess the quality of evidence across studies. In this article, we present the approach applied in these WHO/ILO systematic reviews for performing such assessments on studies of prevalence of exposure. It is called the Quality of Evidence in Studies estimating Prevalence of Exposure to Occupational risk factors (QoE-SPEO) approach. We describe QoE-SPEO’s development to date, demonstrate its feasibility reporting results from pilot testing and case studies, note its strengths and limitations, and suggest how QoE-SPEO should be tested and developed further.

**Methods:**

Following a comprehensive literature review, and using expert opinion, selected existing quality of evidence assessment approaches used in environmental and occupational health were reviewed and analysed for their relevance to prevalence studies. Relevant steps and components from the existing approaches were adopted or adapted for QoE-SPEO. New steps and components were developed. We elicited feedback from other systematic review methodologists and exposure scientists and reached consensus on the QoE-SPEO approach. Ten individual experts pilot-tested QoE-SPEO. To assess inter-rater agreement, we counted ratings of expected (actual and non-spurious) heterogeneity and quality of evidence and calculated a raw measure of agreement (*P_i_*) between individual raters and rater teams for the downgrade domains. *P_i_* ranged between 0.00 (no two pilot testers selected the same rating) and 1.00 (all pilot testers selected the same rating). Case studies were conducted of experiences of QoE-SPEO’s use in two WHO/ILO systematic reviews.

**Results:**

We found no existing quality of evidence assessment approach for occupational exposure prevalence studies. We identified three relevant, existing approaches for environmental and occupational health studies of the *effect* of exposures. Assessments using QoE-SPEO comprise three steps: (1) judge the level of expected heterogeneity (defined as non-spurious variability that can be expected in exposure prevalence, within or between individual persons, because exposure may change over space and/or time), (2) assess downgrade domains, and (3) reach a final rating on the quality of evidence. Assessments are conducted using the same five downgrade domains as the Grading of Recommendations Assessment, Development and Evaluation (GRADE) approach: (a) risk of bias, (b) indirectness, (c) inconsistency, (d) imprecision, and (e) publication bias. For downgrade domains (c) and (d), the assessment varies depending on the level of expected heterogeneity. There are no upgrade domains. The QoE-SPEO’s ratings are “very low”, “low”, “moderate”, and “high”. To arrive at a final decision on the overall quality of evidence, the assessor starts at “high” quality of evidence and for each domain downgrades by one or two levels for serious concerns or very serious concerns, respectively. In pilot tests, there was reasonable agreement in ratings for expected heterogeneity; 70% of raters selected the same rating. Inter-rater agreement ranged considerably between downgrade domains, both for individual rater pairs (range *P_i_*: 0.36–1.00) and rater teams (0.20–1.00). Sparse data prevented rigorous assessment of inter-rater agreement in quality of evidence ratings.

**Conclusions:**

We present QoE-SPEO as an approach for assessing quality of evidence in prevalence studies of exposure to occupational risk factors. It has been developed to its current version (as presented here), has undergone pilot testing, and was applied in the systematic reviews for the WHO/ILO Joint Estimates. While the approach requires further testing and development, it makes steps towards filling an identified gap, and progress made so far can be used to inform future work in this area.

## Graphical abstract

**Figure:**
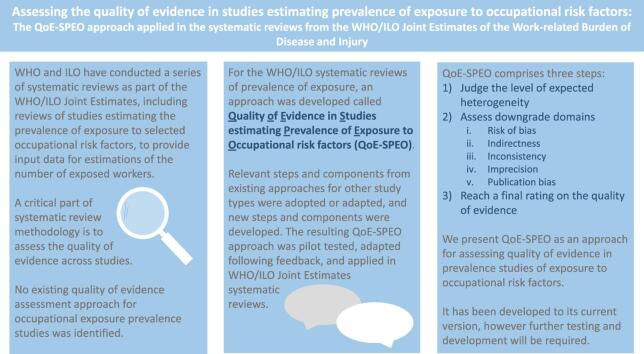

## 1 Background

The World Health Organization (WHO) and International Labour Organization (ILO) have produced the WHO/ILO Joint Estimates of the Work-related Burden of Disease and Injury (WHO/ILO Joint Estimates), supported by a large number of individual experts (Pega et al., 2021a, Pega et al., 2021b, ILO, 2021b, WHO, 2021a). These organizations have produced global, regional and national estimates of exposure to selected occupational risk factors (exposure models) and, consecutively, of the burdens of selected diseases and injuries attributable to these exposures (burden of disease models) (see, for example, Pega et al. (2021a)). They have conducted systematic reviews and meta-analyses of input data for estimating the burden of pairs of occupational risk factors and health outcomes, whose global burdens of disease have never previously been estimated (Descatha et al., 2018, Godderis et al., 2018, Li et al., 2018, Mandrioli et al., 2018, Hulshof et al., 2019, Paulo et al., 2019, Rugulies et al., 2019, Teixeira et al., 2019, Tenkate et al., 2019, Descatha et al., 2020, Li et al., 2020, Pega et al., 2020a, Hulshof et al., 2021a, Hulshof et al., 2021b, Pachito et al., 2021, Rugulies et al., 2021, Teixeira et al., 2021b, Teixeira et al., 2021a, World Health Organization, 2021b). An overview of this series of systematic reviews and its systematic review methodological innovations is provided elsewhere (Pega et al. 2021c).

To estimate the burden of disease attributable to exposure to a risk factor, it is crucial to estimate how widespread (or prevalent) exposure to the risk factor is. Five of the WHO/ILO Joint Estimates systematic reviews aimed to synthesise studies estimating prevalence of exposure (in short, “exposure prevalence studies”) to occupational risk factors: ergonomic risk factors, dusts and/or fibres, solar ultraviolet radiation, noise, and long working hours (Descatha et al., 2018, Godderis et al., 2018, Li et al., 2018, Mandrioli et al., 2018, Hulshof et al., 2019, Paulo et al., 2019, Rugulies et al., 2019, Teixeira et al., 2019, Tenkate et al., 2019, Hulshof et al., 2021b, Teixeira et al., 2021b). An example of the research questions addressed by these reviews is: “What is the point prevalence of occupational exposure to noise above a limit of 85 dB(A) among the global general population of workers?” (Teixeira et al. 2021b).

The need for systematic reviews of studies estimating exposure to risk factors is increasingly being recognized (beyond the WHO/ILO Joint Estimates). Through its *Framework for the Use of Systematic Review in Chemical Risk Assessment* (World Health Organization 2021a) WHO has noted that systematic review methods are underdeveloped in the area of exposure assessment and encouraged exposure prevalence systematic reviews. In the United States of America, the National Academies of Sciences, Engineering, and Medicine have encouraged exposure prevalence systematic reviews, and the Environmental Protection Authority is considering conducting such systematic reviews for its health risk assessments (personal communication, Tracey Woodruff). Experts have called for a framework, tools and approaches for conducting such systematic reviews in a comprehensive and transparent way (Pega et al. Forthcoming), including through modification of existing instruments if feasible (National Academies of Sciences Engineering and Medicine 2021).

Despite this recognition of the need for systematic reviews of exposure prevalence studies, to our World Health Organization 2021b knowledge there are no standardized methods for such reviews. Thus, to address this identified gap, WHO and ILO, supported by individual experts, have developed:

• the Risk of Bias in Studies estimating Prevalence of Exposure to Occupational risk factors (RoB-SPEO) tool (Pega et al., 2020b, Momen et al., 2022)).
• the Quality of Evidence in Studies estimating Prevalence of Exposure to Occupational risk factors (QoE-SPEO) approach (presented in this article).

WHO and ILO have sought to ensure that RoB-SPEO and QoE-SPEO are compatible and complementary.

### 1.1 Studies estimating the prevalence of exposure to occupational risk factors

Occupational exposure prevalence studies determine the presence (and often the level or intensity) of an exposure of interest to an occupational risk factor of interest in each individual in the study population or in a representative sample at one particular time point (Porta 2014). The prevalence of exposure to an occupational risk factor is usually measured in a well-defined population, by determining how many sampled participants have been exposed (i.e., exposed) and how many have not (i.e., unexposed). The prevalence is the number of exposed persons (numerator) divided by the total number of persons (i.e., unexposed persons plus exposed persons) (denominator), usually reported as a percentage. There are several different types of prevalence (Table 1).

**Table 1:** Types of prevalence and their definitions, from Porta (2014).

**Type (*sub-type*)**	**Definition**
Point prevalence	The proportion of individuals with a disease or an attribute at a specified point in time
Period prevalence	The proportion of individuals with a disease or an attribute at a specified period of time. To calculate a period prevalence, the most appropriate denominator for the period must be found
*One-year prevalence*	*The proportion of individuals with the disease or condition at any time during a calendar year. It includes cases arising before and during the year*
*Annual prevalence*	*The proportion of individuals with the disease or attribute at any time during a year. It includes cases of the disease arising before but extending into or through the year as well as those having their inception during the year. Only occasionally used*
*Lifetime prevalence*	*The proportion of individuals who have had the disease or condition for at least part of their lives at any time during their lifecourse*

Studies estimating prevalence are distinct from studies estimating incidence (“the number of new health-related events in a defined population within a specified period of time” (Porta 2014)) or studies estimating prognosis (“the likelihood of future health outcomes in people with a given disease or health condition or with particular characteristics” (p1) (Iorio et al. 2015)). Prevalence studies can be cross-sectional or longitudinal, whereas incidence studies are always longitudinal. Prevalence studies (as defined here) are purely empirical, whereas prognostic studies are predictive modelling studies (sometimes based on empirical data). Studies estimating the prevalence of exposure to occupational risk factors also differ from studies estimating the effect of an occupational health or safety intervention or the effect of an occupational risk factor and health outcome. However, prevalence studies can take multiple designs. As here defined, they could include studies of effect of exposure to a risk factor on a health outcome, if the study also reports prevalence of the risk factor.

Exposure is the “proximity and/or contact with a source of a disease agent (or hazard) in such a manner that effective transmission of the agent or harmful effects of the agent may occur” (Porta 2014). There are several concepts, terms and definitions related to exposure, and these can be related to the different prevalence types (Table 2).

**Table 2:** Concepts, terms and definitions related to exposure, from ES21 Federal Working Group on Exposure Science (2015).

**Concept (*sub-concept*)**	**Definition**	**Related prevalence type(s)**
Exposure	Contact between an agent and a target. Contact takes place at an exposure surface over an exposure period	Point prevalence, period prevalence
*Acute exposure*	*A contact between an agent and a target occurring over a short time, generally less than a day. (Other terms, such as “short-term exposure” and “single dose,” are also used)*	*Point prevalence, period prevalence*
*Chronic exposure*	*A continuous or intermittent long-term contact between an agent and a target. (Other terms, such as “long-term exposure,” are also used)*	*Period prevalence*
*Cumulative exposure*	*The sum of exposures of an organism to a pollutant over a period of time*	*Period prevalence*
*Time averaged exposure*	*The time-integrated exposure divided by the exposure duration. An example is the daily average exposure of an individual to carbon monoxide. (Also called time-weighted average exposure)*	*Period prevalence*
*Time integrated exposure*	*The integral of instantaneous exposures over the exposure duration. An example is the area under a daily time profile of personal air monitor readings, with units of concentration multiplied by time*	*Period prevalence*
Dose	The amount of agent that enters a target after crossing an exposure surface	Point prevalence, period prevalence
*Cumulative dose*	*The total dose resulting from repeated exposures of ionizing radiation to an occupationally exposed worker to the same portion of the body, or to the whole body, over a period of time*	*Period prevalence*

Exposures to occupational risk factors are biological, chemical, physical, ergonomic, mechanical and psychosocial exposures among workers at their workplace, posing a risk known to be harmful to human health (Ott et al. 2007). One example is workplace exposure to crystalline silica dusts, which are an established chemical risk factor for lung cancer among workers (IARC 2009). Both exposure assessment and exposure assignment (the assessment of an exposure based on its determinants including agent, ventilation and worker or environmental characteristics (Burdorf 2005)) are complex for occupational risk factors.

One key and new concept for systematic review of studies estimating prevalence of exposure to occupational risk factors is “expected heterogeneity” (for the definition see Table 3). Exposure status (whether a worker is exposed or unexposed, or exposed above or below a specific exposure limit) and exposure level (the exposure dose or intensity received, expressed in exposure concentration, amount or consideration) can be expected to vary within the same worker over time, and between different workers in the same occupation (Burdorf 2005). They change as workers’ tasks, activities, work processes and work locations change (Burdorf 2005). This “expected heterogeneity” is therefore an important concept to consider when reviewing exposure prevalence studies. The concept of expected heterogeneity is also different to the concepts of heterogeneity and inconsistency as defined in the Grading of Recommendations Assessment, Development and Evaluation (GRADE) approach (Guyatt et al. 2011) (Table 3).

**Table 3:** Comparison of the new concept of “expected heterogeneity” (as used in the QoE-SPEO approach) to the concepts of “heterogeneity” and “inconsistency” (as used in the GRADE approach).

**Concept**	**Definition**	**Relevant study types**
Expected heterogeneity	Real and non-spurious heterogeneity (i.e., variability) that can be expected in the prevalence of exposure, within or between individual persons, because exposure to the risk factor may change over space and/or time	Studies of the *prevalence* of exposure to a risk factor
Heterogeneity	A broad term that can be used to describe any kind of variability among studies in a systematic review (Higgins et al. 2021)	All study types may display heterogeneity
Inconsistency	Differences in relative effect sizes across subgroups. Large inconsistency requires a search for an explanation (“explained heterogeneity”) (Guyatt et al. 2011)	Studies of the *effect* of an exposure on an outcome

### 1.2 Rationale for the development of a new approach for assessing quality of evidence

**Fig. 1:**
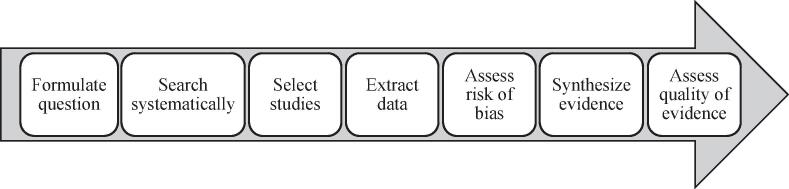
Steps of the systematic review process (p3 in (Pega et al. 2020b)).

Quality of evidence “indicates the extent to which one can be confident that an estimate […] is correct” (p2) (Atkins et al. 2004). Quality of evidence assessments at the level of the entire body of evidence for each outcome are an essential part of the systematic review process (Fig. 1).

#### 1.2.1 Existing methods for assessing quality of evidence in studies of effect

Several methods exist for assessing quality of evidence in *studies of the effect of exposure to an environmental or occupational risk factor on a health outcome* (Krauth et al., 2013, Mandrioli and Silbergeld, 2016, Vandenberg et al., 2016, Whaley et al., 2016, Wang et al., 2019). This includes the Navigation Guide (Woodruff and Sutton, 2014, Lam et al., 2016a, Lam, 2016b) and the Office of Health Assessment and Translation (OHAT) methods (Office of Health Assessment and Translation, 2019, Rooney et al., 2014, Office of Health Assessment and Translation, 2015). Both are based on the Grading of Recommendations Assessment, Development and Evaluation (GRADE) approach (Guyatt et al. 2011) that Cochrane adopts (Higgins et al. 2021). However, there are currently none specifically assessing the quality of evidence in *prevalence studies of exposure to occupational risk factors*. This gap limits health policy and practice: it challenges evidence-based health risk assessments (International Programme on Chemical Safety 2004), in which exposure assessment is a key step (International Programme on Chemical Safety and Inter-Organization Programme for the Sound Management of Chemicals 2010), because assessors do not have a way to evaluate quality of evidence during exposure assessment. The existing methods used by researchers that assess quality in *studies of effect* cannot be directly applied to assess quality of evidence in *occupational exposure prevalence studies* for several reasons detailed in the following three sections.

##### 1.2.1.1 Differences in focus

Occupational exposure prevalence studies are designed to produce estimates of the prevalence of occupational exposures, not test for the effect of occupational exposures on health outcomes (or association). Consequently, prevalence studies investigate neither health outcomes, nor effects, and therefore, methods in existing approaches for assessing related quality of evidence are not applicable.

##### 1.2.1.2 Differences in evidence streams

Evidence regarding *the effect of an exposure* on a health outcome may come from evidence streams other than just human data (here defined as data on human exposures collected using personal samples or other reliable methods). Conversely, evidence on the *prevalence of (human) exposure* to an occupational risk factor comes exclusively from data based on human studies. Therefore, while methods for assessing quality of evidence in studies of health effects of exposure to occupational risk factors need to be able to assess quality of evidence across evidence streams, methods for assessing quality of evidence in prevalence studies need to consider human-based data only.

##### 1.2.1.3 Expected heterogeneity present in exposure prevalence studies

A further reason that existing approaches cannot be applied is the central role that “expected heterogeneity” plays in prevalence studies (Table 3). This concept of expected heterogeneity is different to that of “inconsistency” (or “explained heterogeneity”), which is included in GRADE (Guyatt et al. 2011) and the National Toxicology Program’s Handbook for Preparing Report on Carcinogens Monographs (National Toxicology Program 2015). When using GRADE to assess quality of evidence, reviewers should consider downgrading when i) point estimates vary widely across studies, ii) confidence intervals show minimal or no overlap, iii) the statistical test for heterogeneity suggests statistical significance, and iv) the *I^2^* (quantifying the proportion of variation in relative risks due to among-study differences) is large (Guyatt et al. 2011). The National Toxicology Program handbook details the possible explanations for observed heterogeneity, when considering the effect of carcinogens on cancer risk e.g. differences in study quality or the periods covered (Office of Health Assessment and Translation, 2019, National Toxicology Program, 2015).

While inconsistency can be a reason to downgrade a quality of evidence rating, expected heterogeneity is not. When a body of evidence on prevalence estimates is expected to be heterogeneous, its quality of evidence assessment should not be downgraded if is found to be heterogeneous empirically (as occurs in the GRADE (Guyatt et al. 2011), Navigation Guide (Lam et al., 2016a, Lam, 2016b), and OHAT (Office of Health Assessment and Translation, 2019) approaches). Conversely, if it is expected to be homogeneous (i.e., non-variable) but is found to be heterogeneous empirically, then an assessor may downgrade the quality of evidence. In addition, when assessing studies of exposure prevalence, assessment of downgrade domains other than heterogeneity (such as the domain of imprecision) should depend on the level of expected heterogeneity. For example, a body of prevalence estimates expected to be highly heterogeneous would also be expected to be more imprecise, empirically and statistically, than if homogeneity had been expected. In GRADE, explained heterogeneity observed in the body of evidence affects only the domain of inconsistency (Guyatt et al. 2011). To our knowledge, the QoE-SPEO is the first quality of evidence assessment approach to incorporate expected heterogeneity.

### 1.3 Development of a new approach for assessing quality of evidence

Our objective was to develop a valid and reliable approach for assessing the quality of evidence in prevalence studies of exposure to occupational risk factors. The target audience is researchers and practitioners who want to make such assessments. Ideally, such an approach should:

• provide structured and clear guidance to assessors in plain language;
• enable comprehensive assessment with domains for all important aspects of the quality of evidence;
• allow assessment of studies of any non-randomized design that estimate the prevalence of exposure to occupational risk factors among humans;
• enable differentiated assessment with ratings along defined and unambiguous criteria; and
• enable assessors to systematically, transparently and comprehensively record, document and justify their assessment, including the selected rating and the rationale for its selection.

In addition, it is important that this approach is compatible with and complementary to RoB-SPEO (Pega et al. 2020b), which is currently the only risk of bias tool specifically for assessing exposure prevalence studies. The approach should also produce assessments that adhere to the Guidelines for Accurate and Transparent Health Estimates Reporting (GATHER) (Stevens et al. 2016).

Most existing approaches are organized in steps, which comprise components. For the step “Assess downgrade domains” for example, components in existing approaches include downgrade domains, downgrade considerations, downgrade criteria and downgrade ratings. While they cannot simply be applied in their entirety to assess occupational exposure prevalence studies, applicability of components of existing approaches was considered. This followed the recommendation from the National Academies of Sciences, Engineering, and Medicine, United States of America, to modify existing instruments in developing systematic review methods for exposure prevalence studies if feasible (National Academies of Sciences Engineering and Medicine 2021).

In this article we describe the development process to date, and present the QoE-SPEO approach in its current version (v.4), which includes improvements based on user feedback after it was applied (in its third version) in the WHO/ILO Joint Estimates, as the approach applied for assessing quality of evidence in prevalence studies of exposure to occupational risk factors in the series of WHO/ILO systematic reviews (Descatha et al., 2018, Godderis et al., 2018, Li et al., 2018, Mandrioli et al., 2018, Hulshof et al., 2019, Paulo et al., 2019, Rugulies et al., 2019, Teixeira et al., 2019, Tenkate et al., 2019, Hulshof et al., 2021b, Teixeira et al., 2021b). Our description and considerations may aid other researchers who also wish to assess quality of evidence of exposure prevalence studies. We report results from applying QoE-SPEO v.3 and discuss the potential strengths and limitations of the approach. We recognise that further testing is required to inform future development and discuss some of the potential next steps for further approach development.

## 2 Methods

**Fig. 2:**
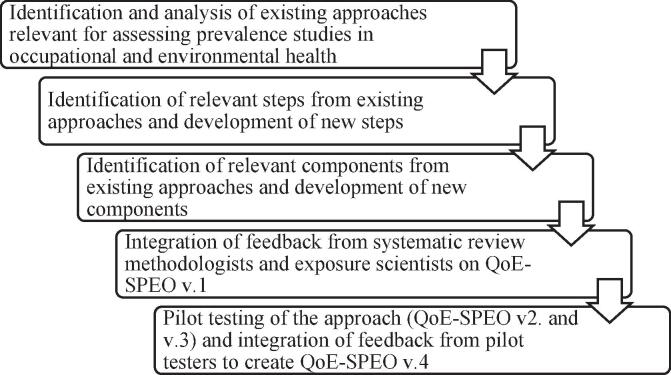
Development of the QoE-SPEO approach to date.

WHO and ILO developed QoE-SPEO with the support of a WHO/ILO Working Group of individual experts on systematic review, environmental and occupational health, and exposure science. Currently QoE-SPEO has been developed up to v.4; the steps taken so far in its development process are summarized in Fig. 2, and each step is described in more detail below. The process of developing QoE-SPEO to its current version paralleled that for RoB-SPEO (Pega et al. 2020b).

### 2.1 Identification and analysis of existing approaches

In June 2018, we undertook a comprehensive, but non-systematic, purposive review of the literature in the electronic bibliographic databases Ovid Medline and EMBASE, to identify and select existing quality of evidence approaches that might be relevant to assessing prevalence studies of exposure to occupational risk factors.

We included approaches:

• focusing on prevalence studies, incidence studies, prognostic studies, studies on the effect of exposure to occupational risk factors on health outcomes, and/or studies of the effect of occupational health or safety interventions on health outcomes;
• for assessing the quality of evidence at the level of the entire body of evidence;
• for assessing quality of evidence in non-randomized studies of human data, because pertinent prevalence studies for our exposure systematic reviews are of this type; or
• based on checklists and/or quantitative scoring (conscious that scoring is not recommended in GRADE or Cochrane due to inherent biases and judgements in scoring).

We excluded approaches:

• for assessing quality of single studies;
• specifically designed for randomized study designs, and in vitro, in vivo and/or mechanistic evidence streams; or
• designed for purposes other than assessing quality of the body of evidence (such as risk of bias tools and reporting guidelines).

Thirty experts in systematic review methods, environmental health, occupational health, occupational safety, and exposure science were also asked to identify relevant approaches. Each expert was a contributor to one or more systematic reviews for the development of the WHO/ILO Joint Estimates (Descatha et al., 2018, Godderis et al., 2018, Li et al., 2018, Mandrioli et al., 2018, Hulshof et al., 2019, Paulo et al., 2019, Rugulies et al., 2019, Teixeira et al., 2019, Tenkate et al., 2019, Descatha et al., 2020, Li et al., 2020, Pega et al., 2020a, Hulshof et al., 2021a, Hulshof et al., 2021b, Pachito et al., 2021, Rugulies et al., 2021, Teixeira et al., 2021a, Teixeira et al., 2021b).

### 2.2 Identification and development of steps

The identified existing approaches were screened for steps potentially relevant for QoE-SPEO. We applied the same criteria for including steps from existing approaches in QoE-SPEO, adopted or adapted, if feasible, as we used when we developed RoB-SPEO (Pega et al. 2020b). Steps were included, if applicable (i.e., either directly or after modification) to:

• non-randomized studies;
• studies estimating any type of prevalence (as defined in Table 1) or incidence;
• studies on human data;
• assessment of exposure (as defined in Table 2); or
• assessment at the level of the entire body of evidence.

Steps were excluded if they were exclusively applicable to:

• randomized studies;
• statistical or mathematical modelling studies;
• studies estimating the effect of an intervention;
• studies estimating the effect of occupational or other exposure;
• studies on in vitro, in vivo, animal and/or mechanistic data;
• assessment of a health outcome;
• assessment of single studies;
• assessment not based on personal judgment; or
• comparison to an ideal target intervention or exposure.

We also identified the need for an additional step to assess expected heterogeneity (see Section 1.2.1.3), which we considered crucial for QoE-SPEO but that did not appear in any existing approach.

### 2.3 Identification and development of components

We analysed the existing approaches, first mapping their components and then identifying the components that were relevant for the QoE-SPEO approach. The RoB-SPEO tool (Pega et al. 2020b) was also screened for potentially relevant components to ensure synergy and match between this tool and QoE-SPEO.

Components identified as relevant were adopted into QoE-SPEO. Most commonly, these were not fit for adoption for QoE-SPEO, requiring either substantial revision or a complete rewrite. For example, downgrading considerations for inconsistency and imprecision needed to take into account the level of expected heterogeneity in the prevalence; we therefore wrote two separate sets of considerations for assessing bodies of evidence on prevalence (i) with expected heterogeneity judged as “high” or “moderate” and (ii) with a “low” level of expected heterogeneity.

We also identified gaps in the current approaches and developed new components that were key for QoE-SPEO. We created all three components that formed the new step for assessing the level of expected heterogeneity in the prevalence. We also developed a reporting table for the new component of recording judgments, ratings and/or rationales for grading evidence down. At the end of this process, we had developed an initial prototype approach (QoE-SPEO version v.1).

### 2.4 Integration of feedback

We sought and integrated feedback on QoE-SPEO in three stages (Table 4), from a diverse group of researchers familiar with the theory behind assessing quality and researchers who would apply the tool, as well as experts who would use its outputs, on QoE-SPEO’s content, structure, and formatting. This was used to sequentially develop the approach from the initial prototype (QoE-SPEO v.1) to the current version presented in this article (QoE-SPEO v.4; Appendix A in the Supplementary data). Selected main innovations introduced at each stage are presented in Table 4. Pilot testers and individual experts that applied the approach in the WHO/ILO systematic reviews generally noted that the approach was clear and practical, and they proposed few and minor changes between QoE-SPEO v.2 and v.4.

**Table 4:** Stages of receipt and integration of feedback during the development of QoE-SPEO and main innovations introduced.

**Stage**	**Feedback**	**Main innovations**
1	Two rounds of feedback on QoE-SPEO v.1 received from two WHO experts and five individual experts on systematic review methods and integrated in QoE-SPEO v.2	- Adopted and/or revised steps and components from existing tools - Developed a new step - Developed new components - Introduced concepts, terms and examples from occupational health and safety
2	First round of pilot testing on QoE-SPEO v.2, feedback received from ten pilot testers and integrated in QoE-SPEO v.3	- Made minor editorial changes
3	Second round of pilot testing on QoE-SPEO v.3 received from 20 individual experts in occupational health, occupational safety, and/or exposure science that applied QoE-SPEO v.3 in a systematic review of prevalence studies for the WHO/ILO Joint Estimates and integrated QoE-SPEO v.4	- Reviewed and improved the concept, definition and term for “expected heterogeneity” - Refined considerations and criteria for some downgrade domains - Added definitions of quality of evidence ratings

### 2.5 Pilot testing and approach finalization

After receiving initial feedback on QoE-SPEO v.1 from seven experts and implementing changes, we undertook pilot-testing QoE-SPEO v.2. One aim of this first round of testing was to receive more feedback to further develop the approach. Another aim was to assess inter-rater agreement (for both individual raters and rater teams). There is minimal scientific consensus on which of several existing methods is preferable for calculating inter-rater agreement. Inter-rater agreement ratings are also not necessarily indicators of an approach’s performance, since ratings are explicitly based on individual judgment; therefore, transparency and reaching a consensus are more important (Losilla et al. 2018). We nevertheless felt that agreement between raters would be indicative of the approach being understood similarly by users.

First, from the ongoing WHO/ILO systematic reviews for the WHO/ILO Joint Estimates (Descatha et al., 2018, Godderis et al., 2018, Li et al., 2018, Mandrioli et al., 2018, Hulshof et al., 2019, Paulo et al., 2019, Rugulies et al., 2019, Teixeira et al., 2019, Tenkate et al., 2019), we selected a body of evidence for pilot testing QoE-SPEO v.2. The body of evidence on the prevalence of occupational exposure to ergonomic (or physical) risk factors was chosen, because, at the time of pilot testing, the systematic review (Hulshof et al., 2019, Hulshof et al., 2021b) on this topic was closest to completion and had, therefore, produced the relevant analytical products needed for assessing quality of evidence. The authors of this review provided the body of evidence for assessment. This comprised five studies (Naidoo et al., 2009, Andersen et al., 2016, Eurofound, 2017, Goldberg et al., 2017). We provided pilot testers with the following analytical products:

• all study records, unpublished analyses and/or re-analyses of the included studies;
• table of characteristics (McKenzie et al. 2019) of the included studies;
• tables of risk of bias (Boutron et al. 2020) for the included studies;
• figure of summary of risk of bias (Boutron et al. 2020); and
• pooled prevalence estimates produced using the input data collected in the systematic reviews (now published in Hulshof et al. (2021b)).

Second, ten co-authors of this article (none of whom had previously contributed to QoE-SPEO’s development) pilot tested QoE-SPEO v.2. We listed pilot testers alphabetically by surname and then paired consecutive pilot testers. They were asked to:

1. conduct individual assessments (QoE-SPEO Steps 1 and 2; steps described in Section 3.2);
2. conduct the consolidated assessment with the second pilot tester in their team (QoE-SPEO Step 3);
3. record the outcomes (i.e., ratings and justifications) of the individual and consolidated assessments in a specific Excel recording sheet; and
4. propose revisions to address issues identified and record issues that the team was unable to address.

Third, we preliminarily tested agreement in ratings between pilot testers by step and/or component, using the following metrics:

1. Agreement on ratings of expected heterogeneity between individual raters in QoE-SPEO Step 1: We calculated the proportion of pilot testers who rated the level of expected heterogeneity as: (1) “no or only minor”; (2) “low”; (3) “medium”; and (4) “high”.
2. Agreement on ratings of downgrade domains between individual raters in QoE-SPEO Step 2: We assessed agreement for ratings of QoE-SPEO’s five downgrade domains between the ten pilot testers, using the individual assessments. Ratings were coded into three analytical categories: (1) “no or only minor concerns”, (2) “serious concerns”, and (3) “very serious concerns”. Using established methods (Armijo-Olivo et al., 2012, Hartling et al., 2013, Savovic et al., 2014, Bilandzic et al., 2016, Morgan et al., 2018, Pega et al., 2020b), we calculated a raw score of inter-rater agreement ($P_{i}$), the proportion of all ratings given by all pilot testers to the *j-th* analytical category, using the following formula:

$$P_{i}=\frac{1}{n(n-1)}\sum_{j=1}^{k}n_{\mathrm{ij}}\left(n_{\mathrm{ij}}-1\right)$$

where *i = 1,…k* is the number of domains (here, k = 5); *j = 1,…k* is the number of possible analytical categories (here, k = 3); and *n* = number of assessors for the component (here n = 10). *P_i_* ranges from 0.00 (no two pilot testers chose the same rating) to 1.00 (all pilot testers chose the same rating).

3. Agreement on domains between rater teams in QoE-SPEO Step 3: We replicated the methods used to calculate agreement on ratings of downgrade domains between individual raters (see 2. above) to calculate such agreement between rater teams, using the teams’ consolidated assessments.
4. Agreement on ratings of quality of evidence in QoE-SPEO Step 3: We counted the number of pilot testers who rated the quality of evidence as (1) “very low”; (2) “low”; (3) “moderate”; and (4) “high”.

Finally, we integrated all feedback received from pilot testers, including proposals for revisions to QoE-SPEO v.2, and reports of issues, to develop QoE-SPEO v.3. An additional round of testing of QoE-SPEO v.3 was carried out opportunistically, as feedback on the tool was sought from 20 individual experts in occupational health, occupational safety, and/or exposure science after they applied it in a systematic review of prevalence studies for the WHO/ILO Joint Estimates. Three improvements were made following this round of testing: we added further explanation regarding the concept of expected heterogeneity; we refined considerations and criteria for some downgrade domains; and we added definitions of quality of evidence ratings to provide more information for raters as this was the downgrade domain with poor inter-rater agreement.

### 2.6 Case studies

We conducted two case studies to collect and report reflections from users that applied QoE-SPEO v.4 in WHO/ILO systematic reviews for use in the further development and testing of the approach. All quality of evidence assessors (N = 5) from two of the systematic reviews of the WHO/ILO Joint Estimates (Hulshof et al., 2021b, Teixeira et al., 2021b) were sent a survey by email and asked to provide their reflections on applying the QoE-SPEO approach. All invited survey participants provided responses. These survey respondents had never been involved in QoE-SPEO’s development or pilot testing and used QoE-SPEO for the first time and only in the WHO/ILO systematic review they participated in. The assessors were asked to provide comments on the advantages, disadvantages and possible improvements for each QoE-SPEO step. The responses with users’ reflections were summarized, these summaries were reviewed and validated by the survey participants, and these summaries are presented in this article (see section 4.3), also with view to provide suggestions for potential priorities and next steps for further QoE-SPEO development.

## 3 Results

### 3.1 Existing approaches

No existing approach was identified for prevalence studies (at least not for assessing a body of evidence). We excluded several approaches from our review (e.g., Loney et al., 1998, Munn et al., 2014, Munn et al., 2015, Stevens et al., 2016, Wells et al., 2019). These were designed specifically for assessing study types not relevant to our tool, designed specifically for assessing the quality of single studies, did not base assessments explicitly on personal judgment, and/or were instruments other than quality of evidence assessment approaches. Selected excluded approaches and the rationale for their exclusion are presented in Appendix B in the Supplementary data. The GATHER reporting checklist (Stevens et al. 2016) and RoB-SPEO (Pega et al. 2020b) were outside of our inclusion criteria, but we checked QoE-SPEO components against related GATHER and RoB-SPEO components to ensure optimal harmonization and compatibility (e.g., use of the same definitions, concepts and terms).

We considered three existing approaches for assessing quality of evidence to be most relevant (Table 5): the GRADE approach for interventions (Guyatt et al. 2011), the Navigation Guide approach for assessment of the risk of environmental exposure to potentially harmful substances (Lam et al. 2014) and the OHAT approach to hazard identification (Office of Health Assessment and Translation, 2019). Although there is an application (Morgan et al. 2016) of the GRADE approach for interventions for evaluating studies of the effect of environmental and occupational exposures on health outcomes, we considered the GRADE approach for interventions more relevant for our purposes (Guyatt et al. 2011), because it provides a document with dedicated domains, considerations, criteria and ratings for quality of evidence assessment. However, although relevant and containing some applicable components, we did not judge any existing approach to be directly applicable in its entirety to assessing quality of evidence in prevalence studies of exposure to occupational risk factors (Table 5). Besides WHO (World Health Organization 2021a) and ILO (through the WHO/ILO Joint Estimates), other agencies and individual experts have also identified this gap in systematic review methods, including the National Academies of Sciences, Engineering, and Medicine (National Academies of Sciences Engineering and Medicine 2021), and the Environmental Protection Authority (personal communication, Tracey Woodruff) in the United States of America.

**Table 5:** Existing quality of evidence assessment approaches identified as relevant for the development of QoE-SPEO.

	**Approach**	**Study types assessed**	**Why this approach is not applicable**
1	GRADE approach (Guyatt et al. 2011)	Studies of the effect of interventions on health outcomes	- Has components applicable exclusively to intervention effectiveness, not prevalence of an exposure - Is not tailored to studies estimating prevalence - Is not tailored to occupational health (Morgan et al. 2019) and exposure scientific studies
2	Navigation Guide approach (Lam et al., 2016a, Lam, 2016b)	Studies of the effects/harms of exposure to an environmental or occupational risk factor on a health outcome (and the severity/probability of these effects/harms)	- Has domains applicable exclusively to studies of the effect of exposure to environmental and occupational risk factors on health outcomes, not prevalence of an exposure - Is not tailored to studies estimating prevalence
3	OHAT approach (Office of Health Assessment and Translation, 2019)	Studies of the toxicity of exposure to environmental and occupational risk factors on health outcomes	- Has domains applicable exclusively to studies of the effect of exposure to environmental and occupational risk factors on health outcomes, not prevalence of exposure - Has components applicable exclusively to two or more evidence streams, not just human-based data - Is not tailored to studies estimating prevalence

Footnotes: GRADE - Grading of Recommendations Assessment, Development and Evaluation; OHAT - Office of Health Assessment and Translation.

### 3.2 Steps of QoE-SPEO

From the three approaches deemed relevant (Table 5), we identified four potential steps (Table 6) (three of these were used in all approaches, however the GRADE approach does not use the first step, i.e. identify *a priori* the baseline level at which the quality of evidence assessment commences based on features of study design). We identified two of these four steps to be relevant for QoE-SPEO: (i) assess downgrade domains and (ii) reach a final decision (rating) on the quality of evidence. We, therefore, adopted these and adapted them as necessary (Table 6).

**Table 6:** Potential steps in the quality assessment of a body of evidence of prevalence studies.

**Step**	**Use in existing approaches (step)**	**Use in QoE-SPEO (step)**	**Use of the step in QoE-SPEO**
Judge the level of expected heterogeneity	No	Yes (Step 1)	Step 1 in QoE-SPEO was developed specifically for the approach. The results of the assessment of the level of expected heterogeneity conducted in Step 1 are the basis for QoE-SPEO’s Step 2.
Determine the baseline quality of evidence rating	Yes (first step) ^a^	No	This step is not used in QoE-SPEO. We did not consider features of study designs to centrally determine quality of evidence in a body of prevalence studies. All QoE-SPEO assessments start at “high quality” of evidence.
Assess downgrade domains	Yes (second step)	Yes (Step 2)	Step 2 in QoE-SPEO was adopted from the second step in existing approaches.
Assess upgrade domains	Yes (third step)	No	This step is not used in QoE-SPEO, because we did not identify any relevant upgrade domains.
Reach a final decision (rating) on the quality of evidence	Yes (fourth step)	Yes (Step 3)	Step 3 in QoE-SPEO was adopted from the fourth step in existing approaches.

Footnotes: ^a^ Not a step in GRADE. QoE-SPEO – Quality of Evidence in Studies estimating Prevalence of Exposure to Occupational risk factors

We judged the two other steps used in the existing approaches as irrelevant for QoE-SPEO. Assessors in QoE-SPEO do not need to determine baseline level of quality of evidence, because all assessments start at “high quality” of evidence; we consider all prevalence studies, at least in principle, to be able to produce high-quality prevalence estimates regardless of study design features. However, in some cases in practice, as some exposures are better assessed in certain study designs, assessors can downgrade the quality of evidence for the relevant domain, based on the specific concern the study design leads to. Assessors using QoE-SPEO do not assess upgrade domains, because such upgrading (as currently conceptualised) is applicable only to studies of effect estimates, and the underlying concepts of large effect size, dose–response relationship and confounding do not transfer to prevalence studies.

We added one step in QoE-SPEO not used in the three existing approaches (Table 6): assessment of the level of expected heterogeneity (as conceptualized and defined in Section 1.2.1.3) of the prevalence of exposure to the risk factor. The evaluation of imprecision and inconsistency depend on the level of expected (versus spurious) heterogeneity observed in the body of evidence. We, therefore, arrived at three steps in QoE-SPEO (Table 6).

### 3.3 Components of QoE-SPEO

Of the 12 components identified from the three existing approaches, we selected seven for inclusion in QoE-SPEO Steps 2 and 3 (Table 7). As Step 1 is unique to QoE-SPEO, there were no components from existing approaches that could have been adopted or adapted.

**Table 7:** Potential components for QoE-SPEO.

	**Component**	**Description**	**Use in existing approaches (step)**	**Use in QoE-SPEO (step)**
1	Instructions	Instructions guiding assessors in their quality of evidence assessments	Yes	Yes
2	Considerations for expected heterogeneity	Specific issues for assessors to consider when rating the expected level of genuine heterogeneity of the prevalence	No	Yes (Step 1)
3	Ratings for expected heterogeneity	The standard categories for rating the expected level of genuine heterogeneity of the prevalence	No	Yes (Step 1)
4	Reporting the assessment of expected heterogeneity	Recording judgments, ratings and/or rationales for expected heterogeneity	No	Yes (Step 1)
5	Considerations for initial quality of evidence	The issues that assessors can consider for when determining the initial level of quality of evidence	Yes	No
6	Ratings for initial level of quality of evidence	The standard categories for ratings the initial level of quality of evidence based on key features of study design	Yes	No
7	Downgrade domains	Domain for grading quality of evidence down for concerns in the domain	Yes	Yes (Step 2)
8	Downgrading considerations	Specific issues for assessors to consider when downgrading	Yes	Yes (Step 2)
9	Ratings for downgrading	The standard categories for rating a downgrade domain indicated by level of concern for the domain	Yes	Yes (Step 2)
10	Reporting the assessment of downgrade domains	Table for recording judgments, ratings and/or rationales for grading evidence down	No	Yes (Step 2)
11	Upgrade domains	Domain for grading quality of evidence up	Yes	No
12	Upgrading considerations	Specific issues for assessors to consider when upgrading	Yes	No
13	Ratings for upgrading	The standard categories for rating an upgrading domain	Yes	No
14	Ratings for quality of evidence	The standard categories for rating quality of evidence	Yes	Yes (Step 3)
15	Rating criteria	The specific criteria for choosing ratings	Yes	Yes (Step 3)
16	Reporting the assessment of quality of evidence	Recording judgments, ratings and/or rationales	Yes	Yes (Step 3)

Footnotes: QoE-SPEO – Quality of Evidence in Studies estimating Prevalence of Exposure to Occupational risk factors

We also developed four new components for QoE-SPEO. Three of these were for Step 1. The other was the introduction of reporting tables in Steps 2 and 3.

### 3.4 Description of QoE-SPEO

The QoE-SPEO approach, as developed up to v.4, is presented in full in Appendix A of the Supplementary data. This is the approach that WHO and ILO, supported by a large number of individual experts, applied in their series of systematic reviews to assess the quality of evidence in occupational exposure prevalence studies for the WHO/ILO Joint Estimates (Descatha et al., 2018, Godderis et al., 2018, Li et al., 2018, Mandrioli et al., 2018, Hulshof et al., 2019, Paulo et al., 2019, Rugulies et al., 2019, Teixeira et al., 2019, Tenkate et al., 2019, Hulshof et al., 2021b, Teixeira et al., 2021b).

#### 3.4.1 General instructions

The QoE-SPEO approach provides general instructions for assessors. These introduce the assessor to the aims, overall structure (including steps and components) and standard format of the approach. Importantly, the assessor is instructed to always record their selected rating (or decision) and detailed justification for it for each step, using the relevant recording table provided at the end of each section.

#### 3.4.2 Steps and components

In QoE-SPEO, assessors consecutively undergo the following three steps:

• Step 1: Judge the level of expected heterogeneity.
• Step 2: Assess downgrade domains.
• Step 3: Reach a final decision (rating) on the quality of evidence.

Each step comprises upfront definitions of key concepts related to that step (e.g., the domain of “inconsistency” includes definitions of “expected heterogeneity”, “heterogeneous body of evidence”, “homogenous body of evidence” and “inconsistency”), instructions specific to the step, considerations for assessments, standard ratings, criteria for ratings (if applicable), and a table for recording and documenting the selected rating and the rationale. Some instructions and considerations, especially regarding downgrading in some domains, differ depending on the level of expected heterogeneity.

In Step 1, assessors consider and rate the level of expected heterogeneity of the prevalence of interest, based on factors such as expected within- and between-country heterogeneity, expected within- and between-worker heterogeneity, and expected within- and between-sector heterogeneity. Because some prevalence estimates can be expected to be (non-spuriously) heterogeneous, a body of evidence should not be downgraded if it is found to be (empirically) heterogeneous for prevalence estimates. When prevalence estimates have been pooled, one indicator of high statistical heterogeneity can be a high *I*^*2*^ statistic (Higgins and Thompson 2002). Step 1 is conducted by each assessor individually. They record the selected rating of the level of expected heterogeneity and justify the selection of this rating in the reporting table.

Step 2 involves assessors considering and rating the five downgrade domains:

1. Risk of bias.
2. Indirectness.
3. Inconsistency.
4. Imprecision.
5. Publication bias.

This step is also completed independently for each outcome (i.e., each prevalence, if two or more are reviewed). For each downgrade domain, the assessor can downgrade the quality of evidence for “serious” concern by one level (-1) or “very serious” concern by two levels (-2). The ratings and justification for each downgrade domain are recorded in the tables for this purpose.

In Step 3, each assessor grades the quality of evidence as “high quality”, “moderate quality”, “low quality” or “very low quality”. This step consolidates any downgrading from Step 2, with consensus reached across a rater team. Grading always starts at “high quality” of evidence, with downgrading as appropriate. The final rating with its justification is recorded to ensure transparency.

### 3.5 Inter-rater agreement from pilot testing of QoE-SPEO

We now present the results from the pilot testing of QoE-SPEO v.3.

#### 3.5.1 Ratings of expected heterogeneity

The level of expected heterogeneity was rated by three of the ten individual raters (30%) as “medium” and by seven (70%) as “high”. None rated the level as “none or only minor” or “low”. We judged this to indicate substantial inter-rater agreement. However, this examination of agreement is simple and relies on a small number of raters. Additional testing is needed to establish inter-rater agreement for this step with more certainty.

Agreement between individual raters in ratings of downgrade domains in Step 2 is shown in Table 8. For the five domains, agreement ranged between 0.36 and 1.00:

**Table 8:** Agreement in ratings of downgrade domains between individual raters and rater teams.

**Downgrade domain**		**Agreement between individual raters (n = 10) in Step 2**	**Agreement between rater teams (n = 5) in Step 3**
		**B_i_**	**B_i_**
1	Risk of bias	**0.36**	**0.20**
2	Indirectness	**0.44**	**0.40**
3	Inconsistency	**0.64**	**0.60**
4	Imprecision	**1.00**	**1.00**
5	Publication bias	**0.80**	**1.00**

Low levels of agreement for the Risk of bias domain can be at least partially explained by the fact that this domain is particularly complex to analyse (e.g. risk of bias assessments using RoB-SPEO comprise consideration of eight different types of bias).

#### 3.5.2 Ratings of downgrade domains by rater teams

Agreement between rater teams in ratings of downgrade domains from Step 3 is also displayed in Table 8. In summary, agreement ranged between 0.20 and 1.00. As for agreement between individual raters, agreement for rater teams was relatively poor for the domain of Risk of bias. The consolidation of individual assessments in Step 3 may have improved inter-rater agreement for the domain of Publication bias. However, this analysis was based on sparse data, with additional testing needed.

#### 3.5.3 Ratings of quality of evidence

The quality of evidence was rated as “high” by one of the five rater teams (20%), “moderate” by one team (20%) and “low” by three teams (60%). None of the rater teams rated it as “very low”. These data are too sparse to rigorously analyse. However, the ratings of both “low” and “high” were each ascertained by at least one group. Additional tests with larger counts of ratings are needed.

## 4 Discussion

### 4.1 Comparison of QoE-SPEO with selected existing approaches

The QoE-SPEO approach that is presented here and was applied in the systematic reviews for the WHO/ILO Joint Estimates differs considerably from other quality of evidence assessment approaches in environmental and occupational health. Table 9, Table 10 present comparisons between the GRADE, Navigation Guide, OHAT and QoE-SPEO approaches.

**Table 9:** Comparison of approaches for assessing the quality of a body of evidence.

**Approach**	**Study types assessed**	**Ratings for expected heterogeneity**	**Baseline quality of evidence**	**Downgrade domains**	**Downgrade ratings**	**Upgrade domains**	**Upgrade ratings**	**Ratings of quality of evidence** (see also Table 8)
GRADE approach (Guyatt et al. 2011)	Studies of the effect of an intervention on a health outcome	–	- Randomized studies: High quality - Non-randomized studies: Low quality	Risk of bias Indirectness Inconsistency Imprecision Publication bias	−1 for a serious concern −2 for a very serious concern	Dose-response Strength of effect Residual confounding increases confidence in effect estimate	+1 for evidence of dose–response and for a large effect +2 for evidence for a very large effect	1. High quality 2. Moderate quality 3. Low quality 4. Very low quality
Navigation Guide approach (Lam et al., 2016a, Lam, 2016b)	Studies of the effects/harms and their severity/ probability of exposure to an environmental or occupational risk factor on a health outcome	–	Moderate quality for human observational studies	Risk of bias Indirectness Inconsistency Imprecision Publication bias	−1 for a serious concern −2 for a very serious concern	Dose-response Strength of effect Residual confounding increases confidence in effect estimate	+1 for evidence of dose–response and for a large effect +2 for evidence for a very large effect	1. High quality 2. Moderate quality 3. Low quality
OHAT approach (Office of Health Assessment and Translation, 2019)	Studies of the toxicity of exposure to environmental and occupational risk factors on health outcomes	–	- Four key features^a^ of study design fulfilled: High confidence - Three features: Moderate confidence - Two features: Low confidence - One or no feature: Very low confidence	Risk of bias across studies Unexplained inconsistency Directness and applicability Imprecision Publication bias	−1 for a serious concern −2 for a very serious concern	1. Large magnitude of association or effect 2. Dose response 3. Residual confounding or other related factors that would increase confidence in the estimated effect 4. Cross-species/ population/ study consistency 5. Other		1. High confidence 2. Moderate confidence 3. Low confidence 4. Very low confidence
QoE-SPEO approach (presented in Appendix A in Supplementary data)	Studies of the prevalence of exposure to an occupational risk factor	2. No or only minor expected heterogeneity Low expected heterogeneity Medium expected heterogeneity High expected heterogeneity In Step 1 of QoE-SPEO	High quality Not a separate step in QoE-SPEO	Risk of bias Indirectness Inconsistency ^b^ Imprecision ^b^ Publication bias In Step 2 of QoE-SPEO	−1 for a serious concern −2 for a very serious concern In Step 2 of QoE-SPEO	- (no upgrading)	- (no upgrading)	1. High quality 2. Moderate quality 3. Low quality 4. Very low quality In Step 3 of QoE-SPEO

Footnotes: ^a^ The four features of study design assessed are: (i) controlled exposure; (ii) exposure prior to outcome; (iii) individual outcome data; and (iv) comparison group used. ^b^ Different sets of criteria depending on the judged level of expected heterogeneity of the prevalence of exposure to the occupational risk factor of interest.

GRADE – Grading of Recommendations Assessment, Development and Evaluation; OHAT – Office of Health Assessment and Translation; QoE-SPEO – Quality of Evidence in Studies estimating Prevalence of Exposure to Occupational risk factors.

**Table 10:** Comparison of explanations for quality of evidence ratings.

**Rating**	**GRADE approach**	**Navigation Guide approach**	**OHAT approach**	**QoE-SPEO approach**
High quality (or confidence)	Further research is very unlikely to change our confidence in the estimate of effect.	Unclear	The true effect is highly likely to be reflected in the apparent relationship.	Further research is very unlikely to change our confidence in the estimate of prevalence.
Moderate quality	Further research is likely to have an important impact on our confidence in the estimate of effect and may change the estimate.	Unclear	The true effect may be reflected in the apparent relationship.	Further research is likely to have an important impact on our confidence in the estimate of prevalence and may change the estimate.
Low quality	Further research is very likely to have an important impact on our confidence in the estimate of effect and is likely to change the estimate.	Unclear	The true effect may be different from the apparent relationship.	Further research is very likely to have an important impact on our confidence in the estimate of prevalence and is likely to change the estimate.
Very low quality	We are very uncertain about the estimate of effect.	Not applicable	The true effect is highly likely to be different from the apparent relationship.	We are very uncertain about the estimate of prevalence.

Footnotes:

GRADE – Grading of Recommendations Assessment, Development and Evaluation; OHAT - Office of Health Assessment and Translation; QoE-SPEO – Quality of Evidence in Studies estimating Prevalence of Exposure to Occupational risk factors.

The main differences between QoE-SPEO and the other approaches are:

a) The QoE-SPEO approach assesses the quality of evidence across studies estimating the prevalence of exposure to occupational risk factors, whereas the other existing approaches assess quality of evidence across studies estimating intervention effectiveness, causal effects on (or association with) health outcomes of environmental and occupational risk factors.
b) QoE-SPEO assesses the quality of evidence along domains relevant to non-randomized prevalence studies within the human evidence stream only. The other three tools, on the other hand, facilitate assessment along domains relevant to studies using various evidence streams (including a mechanistic, animal, in vitro, in vivo and human data). However, they can be tailored to assess the human evidence stream only, as in several of the systematic reviews for the WHO/ILO Joint Estimates, including (Hulshof et al., 2021b, Teixeira et al., 2021b).
c) QoE-SPEO includes a step with relevant components that involve judging expected heterogeneity of the prevalence of interest. Expected heterogeneity is a novel concept in quality assessment that is likely to be particularly relevant for assessing exposure prevalence studies. The assessment of some downgrade domains, especially inconsistency and imprecision, need to be considered in light of the level of expected heterogeneity.
d) QoE-SPEO always starts assessments with the rating of “high quality” of evidence, as all study designs have the potential to provide high quality evidence on exposure prevalence to occupational risk factors (at least in principle). The other three approaches determine the baseline level of quality of evidence based on study design.
e) While all four approaches assess the same five downgrade domains, the specific criteria and considerations for downgrading are necessarily quite different between QoE-SPEO and the three existing approaches.
f) While the existing three approaches assess upgrade domains, QoE-SPEO does not, because the upgrade domains of existing approaches are not applicable to prevalence studies.
g) QoE-SPEO adopts the four ratings of quality (or confidence or certainty) of evidence used in GRADE and OHAT, rather than the three Navigation Guide ratings. For rating prevalence studies, we preferred a more granular set of ratings. Our approach minimally modifies the standard explanation for GRADE ratings to suit assessments of evidence on occupational exposure prevalence (Table 10).

### 4.2 QoE-SPEO’s strengths and limitations

#### 4.2.1 Strengths of QoE-SPEO

Strengths of QoE-SPEO include its rigorous development through iterative rounds of feedback from diverse experts on systematic review methods, occupational health and safety, environmental health and, importantly, exposure science. The approach was developed jointly by systematic review methodologists and practitioners who apply evidence synthesis in policy, ensuring that the proposed approach is applicable not only in theory but also feasible in policy practice, including at the global level. QoE-SPEO builds on, and is aligned with, the GRADE framework and also draws on other relevant approaches. It is designed to be used in combination with the RoB-SPEO risk of bias tool and adhere to the GATHER reporting guidelines (Stevens et al., 2016, Pega et al., 2020b). The use of QoE-SPEO for quality of evidence assessments harmonizes the assessment approach of different raters, providing them all with the same criteria and requirements prior to assessments.

#### 4.2.2 Methodological innovations in QoE-SPEO

QoE-SPEO is the first, and currently the only, domain-based approach for assessing quality of a body of prevalence studies. It introduces methodological innovations, including the concept of “expected heterogeneity” of the prevalence. While expected heterogeneity may not be a consideration for other types of studies and therefore not a component in existing approaches, it is key in studies of prevalence of exposure, influencing the assessment of downgrade domains and quality of evidence. A large number of geographically diverse individual experts working within and across different disciplines (e.g., Epidemiology, Exposure Science and Toxicology) has pilot tested and applied QoE-SPEO in systematic reviews for the development of official, global health norms (i.e., the WHO/ILO Joint Estimates). The preliminary results from these tests and real-world applications suggest that QoE-SPEO performs well across most steps and components within steps, achieving an overall good to very good level of inter-rater agreement for almost all domains. Users of the approach have reported to the principal author of this article that QoE-SPEO was comparatively easy and time-efficient to use, with some exceptions; reflections from users are shown below in Section 4.3.

#### 4.2.3 Limitations of QoE-SPEO

Our review of existing approaches for assessing the quality of a body of evidence was not comprehensive. However, we asked several experts to identify existing approaches for assessing quality of evidence in occupational exposure prevalence studies, and as none were identified, we are confident in our conclusion that no such approach currently exists. Assessors may not be familiar with the novel concept of “expected heterogeneity”, but we have tried to mitigate this by explicitly adding its definition in QoE-SPEO. Testing of QoE-SPEO has been limited so far. Feedback was received from 20 individual experts who trialled QoE-SPEO v.3 (not the improved QoE-SPEO v.4 presented in this article). This involved assessment of one specific body of prevalence studies only (studies estimating the prevalence of occupational exposure with ergonomic risk factors, with associated limitations such as all studies relying on subjective exposure measurements), and comprised a relatively small number of raters and rater teams. The data were thus too sparse for robust analysis and conclusions. Inter-rater agreement was poor for the downgrade domain of risk of bias.

We recognise that this approach requires further reflection and development. It should be considered that the reflections from applying the approach shown in Section 4.3 were provided by experts involved in the WHO/ILO Joint Estimates, however those who applied the approach were not involved in its development. To proceed to the next step in QoE-SPEO’s development, feedback from researchers outside of this group and from more varied scientific backgrounds will be essential.

### 4.3 User experience and reflections from applying QoE-SPEO

Assessors from two of the systematic reviews of the WHO/ILO Joint Estimates provided their reflections on applying the QoE-SPEO approach. Box 1 shows a summary of the comments from assessors who worked on the systematic review of the prevalence of occupational exposure to noise (Teixeira et al. 2021b) and Box 2 for assessors who worked on the systematic review of the prevalence of occupational exposure to ergonomic factor (Hulshof et al. 2021b).

Box 1 **Reflections from the WHO/ILO Joint Estimates systematic review on occupational exposure to noise (Teixeira et al. 2021b).**

**Table:** 

**Summary of body of prevalence studies reviewed**
This systematic review included a large number (65) of prevalence studies across a moderate number (28) of countries in all six WHO regions (Africa, Americas, Eastern Mediterranean, Europe, South-East Asia, and Western Pacific). There were four studies of general populations of workers, and 61 studies of worker populations in high-exposure industrial sectors (e.g., construction) or occupations (e.g., construction workers). The occupational risk factor’s definition is perhaps relatively straightforward: any (high) occupational exposure to noise (≥85dBA). The assessors rated the level of expected heterogeneity as “High”.
**Step 1 of QoE-SPEO: judging the level of expected heterogeneity**
The assessors said the “details and examples of health studies of workers” were helpful additions to this step. They felt that “consideration of both within- and between-worker variations in exposure prevalence is an important stipulation”.
They reported the approach would benefit from further explanations of epidemiological terms, “so that researchers from more distant areas of public health can use and understand the instrument”. They said that assessing expected heterogeneity can be complex, especially for a risk factor like noise, for which the entire working population could be exposed: “considering heterogeneity across sectors and job titles may be challenging, when no prior knowledge exists on the population-level”. Rating heterogeneity across the entire body of evidence was challenging (i.e., evidence from both population-based samples comprising various job titles and from specific industrial sectors or occupations with more narrowly defined job characteristics).
Assessors suggested that an explanation be included to illustrate when “expected or real heterogeneity can occur between studies with low and high prevalence”. They also suggested that the instructions can be adapted “for cases when the prevalence of a novel or less studied risk factor in the entire working population is of interest”. Further, they proposed that it may be appropriate to assess heterogeneity separately for evidence from population-based studies (where heterogeneity is expected, at least between workers, and sometimes also within workers) and evidence from studies in specific industry sectors (where less such heterogeneity is expected); or an approach to average across different study designs could be developed.
**Step 2 of QoE-SPEO: assessing downgrade domains**
The assessors felt the instructions and examples for Step 2 were clear and aided understanding. However, they raised several limitations of this step, relating to the following domains:
*Domain of Risk of bias*
The assessors thought that specific consideration should be given to whether the evidence comes from population-based studies, if the risk factor of interest is prevalent in the entire working population: “evidence from those studies should be attributed higher weight” (at least when the target prevalence is that of all workers in the population). They raised the concern that mixing evidence from high-prevalence industry samples and population-based samples could lead to unrealistic overestimation of all-of-population prevalence.
*Domain of Imprecision*
Assessors requested a tentative definition of what can be considered a “narrow” 95% confidence interval. Additionally, they felt the domain would benefit from “suggestions as to how to reconcile epidemiological heterogeneity rated in Step 1 with observed statistical heterogeneity in the meta-analysis of prevalence model”.
*Domain of Publication bias*
Assessors highlighted that, in studies that report both prevalence and effect of a risk factor, the size and statistical significance of the effect are more likely to be “driving publication bias, rather than the prevalence of the risk factor”. In studies of effect, which are commonly used to extract prevalence data from, greater exposure contrast between exposed and unexposed groups of workers is more likely to reveal significant risk of disease due to higher statistical power, and therefore, “studies where the prevalence of a risk factor is not high may be more likely to be published (because of significant findings), rather than studies in which the prevalence of that risk factor is high and the majority of workers are exposed”. They suggested that assessors should be prompted to consider the primary aims of the included studies.
Another caveat in the publication bias domain is that the suggested funnel plot and Egger’s test for asymmetry as means of detecting publication bias are discouraged in meta-analyses of prevalence (Hunter et al. 2014). Moreover, the Eger test in general is not advised for dichotomous outcomes (Page et al. 2021).
**Step 3 of QoE-SPEO: reaching a final decision (rating) on the quality of evidence**
Assessors said the instructions provided for this step were clear and detailed, and recognized that rating the overall quality of evidence is an iterative and transactional process between assessors. Provision of a completed example could help assessors.

Box 2 **Reflections from the WHO/ILO Joint Estimates systematic review on occupational exposure to ergonomic factors (Hulshof et al. 2021b).**

**Table:** 

**Summary of body of prevalence studies reviewed**
The systematic review included a small number (five) of studies from a large number (36) of countries in only two regions (Africa and Europe). The occupational risk factor is complex, defined multi-componentially as one or more of: force exertion, demanding posture, repetitive movement, hand-arm vibration, kneeling or squatting, lifting, and/or climbing. The assessors rated the level of expected heterogeneity as “moderate”.
**Step 1 of QoE-SPEO: judging the level of expected heterogeneity**
Assessors stated that they considered this an important step as it made them consider upfront that prevalence of a risk factor will not always be the same “for all workers working in the same or in different occupations”. Some reported that it was an easy step to perform.
The assessors suggested that this step of the approach could be improved by including “anchor points for assessing the heterogeneity, e.g. sample size, representativeness of subgroups/demographic/age, providing range or 95% CI [confidence intervals] of prevalence rates”, which could provide guidance for assessors.
**Step 2 of QoE-SPEO: assessing downgrade domains**
The reviewers reported that this was a useful step, and was “clear” and “informative”, making it straightforward to use even with minimal experience.
Disadvantages were that selecting ratings can be challenging, especially when taking expected heterogeneity into account. Additionally, considering multiple domains was challenging, in particular when assessments in the domains of Indirectness, Inconsistency, Imprecision and Publication bias can also be influenced by those of the Risk of bias domain, needing to avoid applying a “double” downgrade for the same concern.
To simplify the approach, assessors suggested allowing downgrading by only one level for each domain (rather than two). Further, they suggested the provision of more examples of typical concerns.
**Step 3 of QoE-SPEO: reaching a final decision (rating) on the quality of evidence**
The assessors considered this a necessary step for reaching a final decision and reported that it was easy to follow. They, however, reported some differences in opinion on “low” versus “very low” ratings; one assessor suggested replacing the rating scale with a three-point scale (low/moderate/high).

### 4.4 Further tool testing and development

Additional performance testing of QoE-SPEO is required to improve and develop the tool further. This should include a comprehensive assessment of inter-rater agreement for ratings of expected heterogeneity, downgrade domains and quality of evidence. Indicators of inter-rater agreement by themselves cannot establish epistemological reliability (i.e., that the same methods have consistently been used across raters) (Pega et al. 2020b). Moreover, an approach based on expert judgment cannot necessarily be assumed to be reliable and should not be assessed against quantitative inter-rater agreement alone or even primarily (Pega et al. 2020b). Pilot testing indicated that comprehensive additional testing is required of inter-rater agreement of ratings of expected heterogeneity in Step 1 and especially of ratings of quality of evidence in Step 3. In addition, the ratings of the downgrade domain of risk of bias in Steps 2 and 3 achieved poor agreement between individual raters and rater teams, and consequently, this component requires additional careful analysis and testing. This may include testing whether risk of bias ratings of single studies produced using RoB-SPEO (and/or their presentation) may be an underlying cause of the observed poor agreement on risk of bias ratings across studies in QoE-SPEO; in that point, a performance assessment of RoB-SPEO found that the tool had good inter-rater agreement for all its risk of bias domains (Momen et al. 2022). An evaluation of the performance of QoE-SPEO when used in combination with RoB-SPEO would also be informative. Evaluations are needed of agreement in ratings among a large number of rater teams, rather than individual raters, using suitable metrics such as Fleiss’ kappa (Fleiss 1971) or Krippendorff’s alpha coefficient (Hayes and Krippendorff 2007). Future performance testing should use the latest QoE-SPEO version (v.4) to assess a diverse range of bodies of occupational exposure prevalence studies. In addition, the usefulness of the output (assessment) to end-users should be evaluated. The application of QoE-SPEO in the systematic reviews of prevalence that WHO and ILO have conducted with individual experts for the WHO/ILO Joint Estimates (Descatha et al., 2018, Godderis et al., 2018, Li et al., 2018, Mandrioli et al., 2018, Hulshof et al., 2019, Paulo et al., 2019, Rugulies et al., 2019, Teixeira et al., 2019, Tenkate et al., 2019) will provide additional information to study QoE-SPEO’s performance. As suggested by one of the assessors (Box 1), it may be necessary to carry out subgroup analyses to provide estimates of prevalence of a risk factor for study records that aim to report prevalence, and study records that report prevalence as part of a study on the effect of the risk factor. For risk factors like exposure to occupational noise, for which the whole working population could potentially be considered exposed, publication bias may mean that studies of effect are more likely to be published if there is greater exposure contrast between exposed and unexposed groups of workers, as this is more likely to reveal significant risk of disease due to higher statistical power.

We have identified some areas for consideration when the QoE-SPEO approach is developed further:

• Step 1 (i.e., evaluating the level of expected heterogeneity) is novel and should, therefore, be reviewed and revised as needed. As it should be carried out as a first step, and to conduct this assessment before assessors have knowledge of the evidence base, it may be most appropriate to move this step to the protocol stage of a systematic review and report the assessment in the protocol before the systematic review commences. Assessors may benefit from further examples and guidance regarding the assessment of expected heterogeneity and how this might influence decisions for the downgrade domains. A set of criteria to evaluate this new concept should be developed. Also, this step may need substantial further refinement following more definitional and conceptual research, such as move to a matrix-type assessment of expected heterogeneity by geographic region, industrial sector, sex and/or other determinants of heterogeneity.
• In Step 2, the downgrade domain of risk of bias may require changes to improve inter-rater agreement. For example, regarding the domain of imprecision, since occupational prevalence estimates from single studies and from meta-analyses commonly have highly skewed 95% confidence intervals (e.g., due to ceiling or floor effects), more detailed guidance could be developed to judge imprecision in prevalence estimates with skewed confidence intervals.
• QoE-SPEO’s scope may include mathematical or statistical modelling studies (e.g., studies that model exposure prevalence based on empirical exposure measurement) and studies that use biomarkers for measuring exposure. Dedicated steps and components may need to be developed to assess the quality of evidence in these studies.
• The decision that all bodies of evidence should start at high quality regardless of study design can be reviewed (if considered necessary). While this was based on the judgement that theoretically studies of all designs could be used to measure a prevalent exposure, some designs may be less suited to a specific research question. For example, cross-sectional studies are appropriate for measuring *point* prevalence (as defined in Table 2), but they may be less appropriate for measuring any type of *period* prevalence (Table 2) of an exposure over time. If exposure measurement over time was the question being addressed, assessors would need to consider downgrading a body of evidence comprised mainly of single-time point cross-sectional studies, if they were deemed eligible for inclusion. Future applications of the approach may wish to consider a first step that sets an appropriate starting point based on the research question, the type of prevalence of interest and the most appropriate study design.
• Evidence might emerge that will support the use of upgrade (or other downgrade) domains.

QoE-SPEO will benefit from further development and refinement over time. We welcome comments from those with expertise on the topic and invite organizations and individual experts to test the approach and provide feedback. This will help further tailor and refine tool components. We recognise, however, that the gaps for systematic reviews of studies of prevalence of exposure to occupational risk factors are not limited to the need for an approach to assess quality of evidence. A wider framework is required to help systematic reviewers develop research questions, and assess risk of bias and quality of evidence.

## 5 Conclusions

QoE-SPEO was applied in its third version to assess the quality of bodies of evidence from occupational exposure prevalence studies in a series of harmonized systematic review and meta-analsyes conducted by WHO and ILO, supported by individual experts, for the development of the WHO/ILO Joint Estimates of the Work-related Burden of Disease and Injury (WHO/ILO Joint Estimates). After amending QoE-SPEO based on feedback from the users, we have developed it, up to its fourth iteration, as described in this paper. We propose this as an approach for assessing quality of evidence in prevalence studies of exposure to occupational risk factors and present the considerations made while developing this tool. QoE-SPEO will benefit from further testing and development, particularly also by assessors from other disciplines and backgrounds, but could be applied in its current form in systematic reviews for health risk assessment at the exposure assessment step, in guideline and policy development, and for the production of health estimates. The approach could also be considered for assessment of quality of evidence in prevalence studies of exposure to other human health risk factors (e.g., environmental, behavioural, metabolic and social ones). Applications in other fields need careful consideration and modifications may be needed.

## Financial support

All authors are salaried staff members of their respective institutions. This publication was prepared with financial support to the WHO from the National Institute for Occupational Safety and Health of the Centres for Disease Control and Prevention of the United States of America (Grant 1E11OH0010676-02; Grant 6NE11OH010461-02–01; and Grant 5NE11OH010461-03–00); the German Federal Ministry of Health (BMG Germany) under the BMG-WHO Collaboration Programme 2020–2023 (WHO specified award ref. 70672); the Spanish Agency for International Cooperation (AECID) (WHO specified award ref.71208). The funders had no role in study design, data collection and analysis, decision to publish, or preparation of the manuscript.

## Author contributions

Conceived the idea: FP, Paul Whaley (Associate Editor for Systematic Reviews, *Environment Internationa*l; Lancaster Environment Centre, Lancaster University, United Kingdom)

Searched literature and provided expert opinions on approach selection: FP, DG, RLM

Developed first prototype of approach: FP, DG

Led all aspects of approach development: FP

Contributed to approach development: DG, LB, DM, RLM, SLN

Developed methodology and facilitated pilot testing: FP

Pilot tested the approach: NC, AD, FB, LG, TL, AM, DP, MSP, PS, TT

Analysed data collected in pilot tests: FP

Provided reflections on user experience: HFvM, AMD, KCP, SN, LT

Wrote first draft and led development of article: FP

Contributed to manuscript writing and development: All authors

Revised the manuscript based on comments from co-authors: FP, NCM

## 8 Disclaimer

The authors alone are responsible for the views expressed in this article and they do not necessarily represent the views, decisions or policies of the institutions with which they are affiliated.

## Declaration of Competing Interest

The authors declare that they have no known competing financial interests or personal relationships that could have appeared to influence the work reported in this paper.
