# Supplementary material for: Assessing the quality of evidence in studies estimating prevalence of exposure to occupational risk factors: The QoE-SPEO approach applied in the systematic reviews from the WHO/ILO Joint Estimates of the Work-related burden of disease and Injury
Source: Environ Int. 2022 Mar;161:107136. doi: 10.1016/j.envint.2022.107136 (PMC8885428; doi:10.1016/j.envint.2022.107136)
Supplement: Supplementary Data 1 [file mmc1.docx]

**Supplementary data for the manuscript:**

**Pega et al.: Assessing the quality of evidence in studies estimating prevalence of exposure to occupational risk factors: The QoE-SPEO approach applied in the systematic reviews from the WHO/ILO Joint Estimates of the Work-related Burden of Disease and Injury**

Appendix A: QoE-SPEO approach for grading the quality of a body of studies estimating the prevalence of exposure to occupational risk factors 2

Appendix B: Selected excluded approaches and the rationale for their exclusion 14

Appendix C: Search terms ………………………………………………………………………

References for all appendices 15

# **Appendix A: QoE-SPEO approach for grading the quality of a body of studies estimating the prevalence of exposure to occupational risk factors**

**General instructions**

*Throughout this document, instructions are marked in italics.*

*The QoE-SPEO approach enables you to systematically and comprehensively judge the quality of evidence on the prevalence of exposure to an occupational risk factor (i.e. across a body of individual studies).*

*This document guides you through the QoE-SPEO approach.*

*The QoE-SPEO approach comprises three steps:*

*Step1: Judge the level of expected heterogeneity*

*Step 2: Assess downgrade domains*

*Step 3: Reach a final decision (rating) on the quality of evidence.*

*Detailed instructions for completing each of these steps are provided below.*

*The possible ratings for the overall quality of evidence within a body of evidence are:*

1. *Very low*
2. *Low*
3. *Moderate*
4. *High.*

*For each step, record your rating and a detailed rationale.*

**Step 1: Judge the level of expected heterogeneity**

*In this step, you judge the level of expected heterogeneity that you reasonably anticipate in the prevalence across the entire body of evidence.*

**Definition**

| **Concept** | **Definition** |
| --- | --- |
| Expected heterogeneity | The actual, real and non-spurious variability in the prevalence of exposure within or between individual workers. |

**Considerations**

The prevalence for exposure to some occupational risk factors is expectedly heterogenous (or variable).

Expected heterogeneity (or variability) can occur within individual workers. For example, one study may find a low prevalence, when the prevalence is expected to be low. Another study of the same sample may find a high prevalence, when the prevalence is expected to be high.

Expected heterogeneity can also occur between workers. For example, one study may find a low prevalence in a sample from a population, when the prevalence is expectedly low. Another study, using another sample, may find an expectedly high prevalence.

**Table for reporting your judgment and justifying it**

*In the below recording table, please judge and justify if the prevalence in the target population can be anticipated to be heterogenous.*

| **Question** | **Answer/rating/justification** |
| --- | --- |
| Do you reasonably anticipate the prevalence of the risk factor to be heterogenous? | *Choose one of:*   - *No or only minor expected heterogeneity anticipated.* - *Expected heterogeneity anticipated* |
| If you do anticipate some expected heterogeneity, how would you rate this? | *Choose one of:*   - *High* - *Medium* - *Low* |
| Justify your judgment | *Add your justification* |

**Step 2: Assess downgrade domains**

*Downgrading is the process of grading the quality of evidence down for a substantive concern. A downgrade domain is a specific, defined component of the quality of evidence.*

*You will assess the entire body of evidence on the following domains:*

1. *Risk of bias*
2. *Indirectness*
3. *Inconsistency*
4. *Imprecision*
5. *Publication bias*

*The number of levels by which the body of evidence is downgraded is determined by the level of concern that you have regarding the domain:*

- *Do not downgrade, if you have no or only minor concerns (-0)*
- *For “serious concerns”, downgrade quality of evidence by one level (-1)*
- *For “very serious” concerns, downgrade quality of evidence by two levels (-2).*

*In Step 1, you judged the level of expected heterogeneity (or variability) in the prevalence. Consider this level when you rate for downgrade domains where relevant, especially the domains of “Inconsistency” and “Imprecision”.*

**Downgrade domain 1: Risk of bias**

**Definitions**

| **Concept** | **Definition** |
| --- | --- |
| Risk of bias | The risk of “a systematic error, or deviation from the truth, in results” (Porta 2014). |

**Possible ratings based on your level of concern**

- Do not downgrade, if you have no or only minor concerns (-0)
- For “serious” concerns, downgrade quality of evidence by one level (-1)
- For “very serious” concerns, downgrade quality of evidence by two levels (-2)

**Considerations**

*You have assessed the risk of bias in individual studies using a domain-based risk of bias tool, such as the RoB-SPEO tool (Pegaet al. 2019). You have presented the figure of “Summary of Risk of Bias”.*

*In line with the GRADE framework (Guyattet al. 2011a), please consider the following principles for moving from ratings of risk of bias in individual studies to ratings of the risk of bias of the entire body of evidence:*

1. In deciding on the overall risk of bias, one does not average across studies (for instance if some studies have “low” or “probably low” risk of bias, some “probably high” risk of bias, and some “high” risk of bias, one does not automatically downgrade the quality of evidence by one level (-1) because of an average rating of “serious” risk of bias). Judicious consideration of the contribution of each study is warranted, with a general guide to focus on high-quality studies.
2. The general extent of each study (broadness of the scope, sample size, etc.) should be evaluated. The contribution made will usually reflect a study’s sample size and quality (including validity and accuracy) of exposure assessment. For example, compared with studies with smaller sample sizes and poorer exposure assessment, studies with larger samples and better exposure assessment will contribute more evidence.
3. You should be reasonably confident that there is a substantial risk of bias across most of the body of evidence before you downgrade the quality of evidence for risk of bias.
4. When you downgrade in the risk of bias downgrade domain, you should consider this in the other downgrade domains and avoid “double-counting” for the same underlying issue.

**Table for reporting your rating and justifying it**

| **Choose one rating for risk of bias** | **Justify your rating** |
| --- | --- |
| - *do not downgrade, if you have no or only minor concerns (-0)* - *for “serious” concerns, downgrade quality of evidence by one level (-1)* - *for “very serious” concerns, downgrade quality of evidence by two levels (-2)* | *Add your justification* |

**Downgrade domain 2: Indirectness**

**Definitions**

| **Concept** | **Definition** |
| --- | --- |
| Indirectness | The population and/or exposure of interest do not match the population and/or exposure measured in included studies. |
| Population of interest | The population that your systematic review aimed to capture. |
| Population measured in included studies | The total population captured in your systematic review, comprising the study samples of all individual studies included in your systematic review. |
| Exposure to the occupational risk factor of interest | The exposure that your systematic review aimed to capture. |
| Exposure to the occupational risk factor measured in included studies | The exposures captured in your systematic review, comprising the study samples of all individual studies included in your systematic review. |

**Possible ratings based on your level of concern**

- Do not downgrade, if you have no or only minor concerns (-0)
- For “serious” concerns, downgrade quality of evidence by one level (-1)
- For “very serious” concerns, downgrade quality of evidence by two levels (-2)

**Considerations**

*You do not need to downgrade the quality of evidence when the evidence is direct, meaning that it measures the prevalence of the exposure of interest in the population of interest.*

*However, you need to downgrade for indirectness if there are substantial and meaningful differences between:*

- *the population of interest and the population measured in the included studies (e.g. the population of interest is painters, and the population measured in included studies is painters and construction workers) and/or*
- *between the exposure of interest and the exposure measured in the included studies (e.g. the exposure of interest is occupational exposure with benzene while the study examines the exposure prevalence of generic air pollution).*

*Keep in mind that some differences may be negligible, and some may not be clinically meaningful. In these cases, you should not downgrade the quality of evidence. For example, you may have found a substantial difference in workplace of interest and workplace measured but consider this to not be biologically meaningful.*

*You can consider the following dimensions for assessing indirectness in exposure to the occupational risk factor:*

- 1. Proxy measurement: If an occupation is used as a proxy for exposure to an occupational risk factor, then this may require downgrading of the quality of evidence. However, if the exposure to the occupational risk factor (e.g. exposure to welding fumes) is entirely tied to a defined occupation (e.g. occupation as a welder), downgrading might not be necessary.
  2. Multiple occupational exposure sources: If there are multiple sources from which the worker can be exposed to the occupational risk factor, and not all sources are captured, you may want to downgrade the quality of evidence. For example, if included studies measured occupational noise from one source only (e.g. a hammer) and not from all other sources (e.g. machineries) exposing the worker during the measurement period.
  3. Non-occupational exposure**:** If there are both occupational and non-occupational sources of exposure to the risk factor, and the included studies combined occupational with non-occupational sources in their exposure measurements, you may want to consider downgrading the quality of evidence. For example, for occupational noise exposure, if included studies captured in their exposure measurement the noise level in the factory (i.e., the occupational exposure) and also the noise level from recreational listening to music by headphones (i.e., a non-occupational source).
  4. Multiple occupational exposure pathways**:** If the included studies captured only one of several exposure pathways (e.g. only captured respiratory exposures, when it should have captured respiratory, skin and ingestion exposures), you may want to consider downgrading the quality of evidence.
  5. Less relevant occupational exposure pathways: If the included studies captured a less relevant exposure pathway (e.g., ingestion exposure was captured but respiratory exposure was more relevant), you may want to consider downgrading the quality of evidence. If you downgrade the quality of evidence for non-capture of a relevant pathway, please provide supporting empirical evidence of the pathway’s relevance.
  6. Timing of exposure measurement: If the timing of exposure measurements in included studies does not capture the effective exposure of the worker to the occupational risk factor of interest, then you may want to downgrade the quality of evidence. For example, if an exposure was measured only at the beginning of a work period, even though the significant exposure occurs during the middle of the period, then this may lead to indirect measurements.
  7. Amount of time captured: If the exposure is only measured for a part of the entire working time (e.g. only for four hours out of a total of eight working hours), the exposure assessment may not have captured the exposure well. Season of the year during which exposure was assessed could also be relevant here; assessment of exposure to pesticides outside the spray/growth season may be indirect when the objective is to capture all relevant pesticide exposures.
  8. Point in time of measurement: In cases where the level of exposure to the risk factor is not constant during the period of work, if the measurement was conducted at selected points of time, this could be indirect and not capture the correct exposure level.
  9. Frequency and number of measurements taken: If the level of exposure to the risk factor is not constant during the period of work, but only a small number of measurements were taken, then you may need to downgrade the quality of evidence. Infrequent measurements instead of multiple and frequent measurements during the workday may miss the relevant levels of exposure.
  10. Capture of use of protective gear: If exposure has been measured only when protective or corrective measures were put in place, you may downgrade the evidence if these measures are not the norm. Examples include measurement of exposure when protective gear was being used, even though it is not normally used; or when an exposure is measured only when ventilation was used to remove the risk factor, despite ventilation not always being in place. If exposure was measured outside of respiratory protective device, when the exposure of interest was exposure entering the lungs, you may want to downgrade for indirectness.
  11. Proximity of measure to worker: If measurements are carried out far from the workers, so that the exposure status or level captured do not reflect the actual exposure status or level of the worker (e.g measurement of background (so-called far-field) exposure, as opposed to personal air sampling (i.e. samples taken in the breathing zone), then you may want to downgrade for indirectness.
  12. Exposure via proxy of occupation or job task (job-exposure matrix): If the exposure is assigned based on the worker’s occupation or job task, you may want to downgrade. A job-exposure matrix is a very useful proxy for exposure when studying how occupational risk factors relate to health outcomes; however, for exposure prevalence (only) there may be some elements that need consideration, e.g. how the job-exposure matrix was constructed (by measurement or by expert judgment). Was it constructed as a general-purpose tool or for a very particular/narrow aim and/or to be applied in a particular country or geographical region? In the latter case, the descriptors for jobs may not match the job title(s) (well) and/or the country/countries or geographical region(s) of interest.

**Table for reporting your rating and justifying it**

*If you believe there is a substantial and meaningful difference in population and/or exposure, when you justify your rating, please specify if you believe this difference leads to an underestimate or an overestimate of the exposure.*

| **Choose one rating for risk of bias** | **Justify your rating** |
| --- | --- |
| - *do not downgrade, if you have no or only minor concerns (-0)* - *for “serious” concerns, downgrade quality of evidence by one level (-1)* - *for “very serious” concerns, downgrade quality of evidence by two levels (-2)* | *Add your justification* |

**Downgrade domain 3: Inconsistency**

**Definitions**

| **Concept** | **Definition** |
| --- | --- |
| Expected heterogeneity | The actual, real variability in the prevalence of exposure within or between individual workers. |
| Heterogeneous body of evidence | A body of evidence in which estimates of prevalence are:   - heterogeneous (or variable) within studies; or - heterogeneous between studies; or - heterogeneous within and between studies. |
| Homogenous body of evidence | A body of evidence in which estimates of prevalence are homogenous (or comparable) both within and across studies. |
| Inconsistency | The variability in the prevalence of exposure within and/or between individual workers that is due to factors other than genuine, real variability within and/or between workers. |

**Possible ratings based on your level of concern**

- Do not downgrade, if you have no or only minor concerns (-0)
- For “serious” concerns, downgrade quality of evidence by one level (-1)
- For “very serious” concerns, downgrade quality of evidence by two levels (-2)

**Considerations**

*In Step 1, you have rated the level of expected heterogeneity that you anticipate in the body of evidence. Your rating of the level of expected heterogeneity is important for your assessment of inconsistency (or unexpected heterogeneity) in the body of evidence. Because considerations differ depending on your rating of expected heterogeneity, they are provided separately below.*

**If you rated expected heterogeneity as “low”:**

- *If the body of evidence is homogenous (or comparable) within and between studies, downgrading may not be necessary.*
- *If the body of evidence is heterogenous (or variable) within and between studies, then you may want to downgrade for inconsistency. For example, assume that you anticipated workers in the occupation “welder” to always be exposed to welding fumes, then if the body of evidence is heterogeneous (e.g. between 10% and 100% of welders were exposed across studies), you may want to downgrade the quality of evidence for inconsistency.*

**If you rated expected heterogeneity as “high” or “medium”:**

- *If the body of evidence is homogenous (or comparable) within and between studies, you may want to consider downgrading the quality of evidence.*
- *If the body of evidence is heterogeneous (or variable) within and between studies, downgrading may not be necessary. For example, assume that metal workers are occupationally exposed to different levels of noise, both within worker and between workers (i.e. the noise levels are expectedly variable within and between metal workers), if a study finds large heterogeneity in status (or level) of noise exposure measured among metal workers, you may not need to downgrade the quality of evidence.*

**Table for reporting your rating and justifying it**

| **Choose one rating for inconsistency** | **Justify your rating** |
| --- | --- |
| - *do not downgrade, if you have no or only minor concerns (-0)* - *for “serious” concerns, downgrade quality of evidence by one level (-1)* - *for “very serious” concerns, downgrade quality of evidence by two levels (-2)* | *Add your justification* |

**Downgrade domain 4: Imprecision**

**Definitions**

| **Concept** | **Definition** |
| --- | --- |
| Imprecision | The measure of statistical variability of a prevalence estimate due to random errors (not systematic error or bias). |

**Possible ratings based on your level of concern**

- Do not downgrade, if you have no or only minor concerns (-0)
- For “serious” concerns, downgrade quality of evidence by one level (-1)
- For “very serious” concerns, downgrade quality of evidence by two levels (-2)

**Considerations**

*In Step 1, you rated the level of expected heterogeneity that you anticipate in the body of evidence. Your rating of the level of expected heterogeneity is important for your assessment of imprecision in the body of evidence. Because considerations differ depending on your rating of expected heterogeneity, they are provided separately below.*

**If you rated expected heterogeneity in Step 1 as “low”:**

- *If the body of evidence of prevalence estimates is imprecise, you may want to downgrade for imprecision. If you assume no expected inconsistency in the exposure, and you consider the sample size to be adequately large and the exposure measurement to be adequately accurate and reliable, you would also expect that the standard deviation of the measured exposure should be relatively low and therefore, the confidence intervals around point estimates should be relatively narrow across studies. If you then find wide confidence intervals, this should indicate imprecision in exposure measurement (as opposed to expected inconsistency in the exposure) and you may want to downgrade the quality of evidence.*
- *If the body of evidence of prevalence estimates is precise, downgrading may not be necessary.*

**If you rated expected heterogeneity in Step 1 as “high” or “medium”:**

- *If the body of evidence of prevalence estimates is imprecise, downgrading may not be necessary. If you assume expected heterogeneity in the exposure, then you would also reasonably expect a relatively large standard deviation in the measured exposure across studies and consequently wide confidence intervals around the point estimates of the prevalence across studies or of the pooled prevalence estimate, respectively. So, if you find a wide confidence interval in your measured exposure across studies, you should not downgrade.*
- *If the body of evidence of prevalence estimates is precise, you may have already downgraded the body of evidence for inconsistency; to avoid “double-counting” of concerns for the quality of evidence you may not want to downgrade the body of evidence also for imprecision.*

**Table for reporting your rating and justifying it**

| **Choose one rating for imprecision** | **Justify your rating** |
| --- | --- |
| - *do not downgrade, if you have no or only minor concerns (-0)* - *for “serious” concerns, downgrade quality of evidence by one level (-1)* - *for “very serious” concerns, downgrade quality of evidence by two levels (-2)* | *Add your justification* |

**Downgrade domain 5: Publication bias**

**Definitions**

| **Concept** | **Definition** |
| --- | --- |
| Publication bias | “the publication or non-publication of research findings depending on the nature and direction of the results” (Dickersin and Min 1993; Guyattet al. 2011b) |

**Possible ratings based on your level of concern**

- Do not downgrade, if you have no or only minor concerns (-0)
- For “serious” concerns, downgrade quality of evidence by one level (-1)
- For “very serious” concerns, downgrade quality of evidence by two levels (-2)

**Considerations**

*For studies on the prevalence of exposures to risk factors, journals may be more likely to publish those that overestimate exposure, depending on the specific context and interests at play. If you are concerned that such publication bias may be present in the included studies, you may want to downgrade the quality of evidence. You may also be concerned that studies finding no or very low exposure to the risk factor may be less likely to be published, because journals are less likely to publish null findings, and this may again require that you downgrade the quality of evidence.*

*You may be more concerned about publication bias if the body of evidence consists of:*

- *early studies (or studies that report the prevalence of exposure to the occupational risk factor for the first time);*
- *studies that are small; or*
- *studies with a conflict of interest, such as sponsorship by a relevant industry.*

*If you conduct an empirical examination of patterns of results (e.g., funnel plot tested using Egger’s test for asymmetry) and find suggestion of publication bias, you should interpret results with caution.*

*More compelling than any theoretical exercise would be success in obtaining the results of some unpublished studies and demonstrating that the published and unpublished data show different results.*

*Comprehensive searches of unpublished studies, grey literature and studies published in languages other than English are important, but not sufficient, to address publication bias.*

*If you judge that publication bias is present, when you justify your rating, please indicate if you believe that the bias would lead to an underestimation or an overestimation of exposure.*

**Table for reporting your rating and justifying it**

| **Choose one rating for publication bias** | **Justify your rating** |
| --- | --- |
| - *do not downgrade, if you have no or only minor concerns (-0)* - *for “serious” concerns, downgrade quality of evidence by one level (-1)* - *for “very serious” concerns, downgrade quality of evidence by two levels (-2)* | *Add your justification* |

**Step 3: Reach a final decision (rating) on the quality of evidence**

**All assessors agree on one rating for each downgrade domain**

*Please compile into one document all assessors’ independent assessments for all downgrade domains. An assessment for a downgrade domain comprises (i) the rating and (ii) the justifications for the ratings. You should arrive at one document containing all assessments of all assessors, for each of the five downgrade domains.*

*Share the document with all assessors.*

*Separately for each domain, discuss until you have reached consensus on the overall rating for the domain.*

*If a concern could be considered under two downgrade domains, you should avoid counting the same concern twice. If a serious concern is fully shared by two downgrade domains, you may,*

*want to only downgrade the quality of the body of evidence for this serious concern by one level (-1). If the same serious concern, however, acts independently in each of two downgrade domains (i.e. no double-counting), you may want to downgrade the body of evidence by two levels (-2).*

*For each downgrade domain, rigorously document the final agreed downgrade rating and document in detail the reasons for this downgrade rating, using the table for reporting provided below.*

**Table for all assessors reporting their agreed final downgrade ratings and justifying them**

| **Downgrade domain** | **Choose one final rating** | **Justify your rating** |
| --- | --- | --- |
| Risk of bias | *Choose one of:*   - *do not downgrade, if you have no or only minor concerns (-0)* - *for “serious” concerns, downgrade quality of evidence by one level (-1)* - *for “very serious” concerns, downgrade quality of evidence by two levels (-2)* | *Add your justification* |
| Indirectness | *Choose one of:*   - *do not downgrade, if you have no or only minor concerns (-0)* - *for “serious” concerns, downgrade quality of evidence by one level (-1)* - *for “very serious” concerns, downgrade quality of evidence by two levels (-2)* | *Add your justification* |
| Inconsistency | *Choose one of:*   - *do not downgrade, if you have no or only minor concerns (-0)* - *for “serious” concerns, downgrade quality of evidence by one level (-1)* - *for “very serious” concerns, downgrade quality of evidence by two levels (-2)* | *Add your justification* |
| Imprecision | *Choose one of:*   - *do not downgrade, if you have no or only minor concerns (-0)* - *for “serious” concerns, downgrade quality of evidence by one level (-1)* - *for “very serious” concerns, downgrade quality of evidence by two levels (-2)* | *Add your justification* |
| Publication bias | *Choose one of:*   - *do not downgrade, if you have no or only minor concerns (-0)* - *for “serious” concerns, downgrade quality of evidence by one level (-1)* - *for “very serious” concerns, downgrade quality of evidence by two levels (-2)* | *Add your justification* |

**All assessors reach one final decision (rating) of the quality of evidence**

*Based on the agreed final downgrade ratings, please decide on the rating of the overall quality of evidence.*

*Start at “high” quality of evidence.*

*For each concern judged to be “serious”, downgrade by one level (-1), and for each concern judged to be “very serious”, downgrade by two levels (-2). For example, you may downgrade from “high” quality of evidence to “low” quality of evidence by two levels (-2): from “moderate” for a “serious” concern regarding risk of bias (-1), then to “low” for a “serious” concern regarding imprecision (-1).*

*Rigorously document the final rating of the quality of evidence chosen and document in detail the agreed reasoning, using the table for reporting provided below.*

**Table for all assessors reporting their agreed final rating and justifying it**

| **Choose one final rating of quality of evidence** | **Justify your rating** |
| --- | --- |
| - *Very low quality of evidence: We are very uncertain about the estimate of prevalence.* - *Low quality of evidence: Further research is very likely to have an important impact on our confidence in the estimate of prevalence and is likely to change the estimate.* - *Moderate quality of evidence: Further research is likely to have an important impact on our confidence in the estimate of prevalence and may change the estimate.* - *High quality of evidence: Further research is very unlikely to change our confidence in the estimate of prevalence.* | *Add your justification* |

# **Appendix B: Selected excluded approaches and the rationale for their exclusion**

| **Excluded tool** | **Rationale for exclusion** |
| --- | --- |
| Critical Appraisal Guidelines and Scoring (Loneyet al. 1998) | This approach uses a checklist and scoring to assess the quality of evidence of studies estimating the prevalence of a health outcome at the level of the individual study. However, QoE-SPEO seeks to assess quality of evidence explicitly based on personal judgment (Sterneet al. 2016), assess studies estimating the prevalence of exposure to occupational risk factors specifically, and assess at the level of the entire body of evidence. |
| Joanna Briggs Institute Prevalence Critical Appraisal Tool (Munnet al. 2015; Munnet al. 2014) | This approach uses a checklist and scoring to assess the quality of evidence of studies estimating the prevalence of a health outcome at the level of the individual study. However, QoE-SPEO seeks to assess quality of evidence explicitly based on personal judgment (Sterneet al. 2016), assess studies estimating the prevalence of exposure to occupational risk factors specifically, and assess at the level of the entire body of evidence. |
| Newcastle-Ottawa scale for assessing the quality of non-randomized studies in meta-analyses (Wellset al. 2019) | This tool is for assessing risk of bias and quality of evidence of cohort studies and case-control studies only, and at the level of the individual study. However, QoE-SPEO seeks to assess all prevalence studies of exposure to occupational risk factors and at the level of the entire body of evidence. |
| Guidelines for Accurate and Transparemnt Heath Estimates Reporting (GATHER) (Stevenset al. 2016; The GATHER Working Group 2016) | Although GATHER outlines selected quality of evidence categories for reporting input data, it is a reporting guideline, not an approach for assessing quality of evidence. |

# **Appendix C: Search strategy – Web of Science**

((quality NEAR/3 evidence)

AND

(approach OR tool)

AND

(prevalen*  OR inciden*))

# **References for all appendices**

Dickersin, K.; Min, Y.I. Publication bias: the problem that won't go away. Ann N Y Acad Sci 1993;703:135-146; discussion 146-138

Guyatt, G.; Oxman, A.D.; Akl, E.A.; Kunz, R.; Vist, G.; Brozek, J.; Norris, S.; Falck-Ytter, Y.; Glasziou, P.; DeBeer, H.; Jaeschke, R.; Rind, D.; Meerpohl, J.; Dahm, P.; Schunemann, H.J. GRADE guidelines: 1. Introduction-GRADE evidence profiles and summary of findings tables. J Clin Epidemiol 2011a;64:383-394

Guyatt, G.H.; Oxman, A.D.; Montori, V.; Vist, G.; Kunz, R.; Brozek, J.; Alonso-Coello, P.; Djulbegovic, B.; Atkins, D.; Falck-Ytter, Y.; Williams, J.W., Jr.; Meerpohl, J.; Norris, S.L.; Akl, E.A.; Schunemann, H.J. GRADE guidelines: 5. Rating the quality of evidence--publication bias. J Clin Epidemiol 2011b;64:1277-1282

Loney, P.L.; Chambers, L.W.; Bennett, K.J.; Roberts, J.G.; Stratford, P.W. Critical appraisal of the health research literature: prevalence or incidence of a health problem. Chronic Dis Can 1998;19:170-176

Munn, Z.; Moola, S.; Lisy, K.; Riitano, D.; Tufanaru, C. Methodological guidance for systematic reviews of observational epidemiological studies reporting prevalence and cumulative incidence data. Int J Evid Based Healthc 2015;13:147-153

Munn, Z.; Moola, S.; Riitano, D.; Lisy, K. The development of a critical appraisal tool for use in systematic reviews addressing questions of prevalence. Int J Health Policy Manag 2014;3:123-128

Pega, F.; Norris, S.L.; Backes, C.; Bero, L.A.; Descatha, A.; Gagliardi, D.; Godderis, L.; Loney, T.; Modenese, A.; Morgan, R.L.; Pachito, D.; Paulo, M.B.S.; Scheepers, P.T.J.; Schlunssen, V.; Sgargi, D.; Silbergeld, E.K.; Sorensen, K.; Sutton, P.; Tenkate, T.; Torreao Correa da Silva, D.; Ujita, Y.; van Deventer, E.; Woodruff, T.J.; Mandrioli, D. RoB-SPEO: A tool for assessing risk of bias in studies estimating the prevalence of exposure to occupational risk factors from the WHO/ILO Joint Estimates of the Work-related Burden of Disease and Injury. Environ Int 2019;135:105039

Porta, M. A Dictionary of Epidemiology.6 ed^eds. New York, NY: Oxford University Press; 2014

Sterne, J.A.; Hernan, M.A.; Reeves, B.C.; Savovic, J.; Berkman, N.D.; Viswanathan, M.; Henry, D.; Altman, D.G.; Ansari, M.T.; Boutron, I.; Carpenter, J.R.; Chan, A.W.; Churchill, R.; Deeks, J.J.; Hrobjartsson, A.; Kirkham, J.; Juni, P.; Loke, Y.K.; Pigott, T.D.; Ramsay, C.R.; Regidor, D.; Rothstein, H.R.; Sandhu, L.; Santaguida, P.L.; Schunemann, H.J.; Shea, B.; Shrier, I.; Tugwell, P.; Turner, L.; Valentine, J.C.; Waddington, H.; Waters, E.; Wells, G.A.; Whiting, P.F.; Higgins, J.P. ROBINS-I: a tool for assessing risk of bias in non-randomised studies of interventions. Bmj 2016;355:i4919

Stevens, G.A.; Alkema, L.; Black, R.E.; Boerma, J.T.; Collins, G.S.; Ezzati, M.; Grove, J.T.; Hogan, D.R.; Hogan, M.C.; Horton, R.; Lawn, J.E.; Marusic, A.; Mathers, C.D.; Murray, C.J.; Rudan, I.; Salomon, J.A.; Simpson, P.J.; Vos, T.; Welch, V. Guidelines for Accurate and Transparent Health Estimates Reporting: the GATHER statement. Lancet 2016;388:e19-e23

The GATHER Working Group. The GATHER Statement: Explanation and Elaboration. Geneva: World Health Organization; 2016

Wells, G.; Shea, B.; O'Connell, D.; Peterson, J.; Welch, V.; Losos, M.; Tugwell, P. The Newcastle-Ottawa Scale (NOS) for assessing the quality of nonrandomised studies in meta-analyses. Ottawa, Canada: Ottawa Hospital Research Institute; 2019
